# Shortcuts for biomonitoring programs of stream ecosystems: Evaluating the taxonomic, numeric, and cross-taxa congruence in phytoplankton, periphyton, zooplankton, and fish assemblages

**DOI:** 10.1371/journal.pone.0258342

**Published:** 2021-10-14

**Authors:** Ruan Carlos Pires Faquim, Karine Borges Machado, Fabrício Barreto Teresa, Pedro Henrique Francisco de Oliveira, Gustavo Fernandes Granjeiro, Ludgero Cardoso Galli Vieira, João Carlos Nabout

**Affiliations:** 1 Câmpus Anápolis de Ciências Exatas e Tecnológicas—Henrique Santillo, Universidade Estadual de Goiás, Anápolis, Goiás, Brazil; 2 Departamento de Ecologia, Instituto de Ciências Biológicas, Universidade Federal de Goiás, Goiânia, Goiás, Brazil; 3 Faculdade de Planaltina, Universidade de Brasília, Planaltina, Distrito Federal, Brazil; Universidade Federal de Mato Grosso do Sul, BRAZIL

## Abstract

Different biological groups can be used for monitoring aquatic ecosystems because they can respond to variations in the environment. However, the evaluation of different bioindicators may demand multiple financial resources and time, especially when abundance quantification and species-level identification are required. In this study, we evaluated whether taxonomic, numerical resolution and cross-taxa can be used to optimize costs and time for stream biomonitoring in Central Brazil (Cerrado biome). For this, we sampled different biological groups (fish, zooplankton, phytoplankton, and periphyton) in stream stretches distributed in a gradient of land conversion dominated by agriculture and livestock. We used the Mantel and Procrustes analyses to test the association among different taxonomic levels (species to class), the association between incidence and abundance data (numerical resolution), and biological groups. We also assessed the relative effect of local environmental and spatial predictors on different groups. The taxonomic levels and numerical resolutions were strongly correlated in all taxonomic groups (r > 0.70). We found no correlations among biological groups. Different sets of environmental variables were the most important to explain the variability in species composition of distinct biological groups. Thus, we conclude that monitoring the streams in this region using bioindicators is more informative through higher taxonomic levels with occurrence data than abundance. However, different biological groups provide complementary information, reinforcing the need for a multi-taxa approach in biomonitoring.

## Introduction

The biodiversity of aquatic freshwater ecosystems is of great local and global importance, which has experienced more pronounced declines than terrestrial environments [1]. Besides, aquatic environments are linked directly to peripheral terrestrial ecosystems. Therefore, structural changes in terrestrial environments [2, 3] can promote changes in the physical-chemical characteristics of the water and physical structure of the aquatic habitat [4], negatively affecting the aquatic biodiversity [5–7]. For this reason, urgent actions are necessary to protect freshwater ecosystems [8], in addition to methods that improve the assessment and biomonitoring of aquatic biodiversity [9].

Biomonitoring assessments often use bioindicator species or communities with specific requirements, together with a set of chemical and physical variables of the environment. Thus, changes in these variables can lead to changes in the species presence-absence and abundance, besides morphological, physiological, and behavioral interferences, or even local extinction [10]. Usually, biomonitoring research and programs focus on a single or a set of taxonomic groups. Thus, phytoplankton [11–13], periphyton [14–16], zooplankton [13, 17, 18], macroinvertebrates [19], and fish [20, 21], can be used alone or together with other taxonomic groups to assess the responses of different levels of environmental degradation in aquatic ecosystems [22].

Aspects related to implementation, such as low-cost sampling and identification and simplification of protocols, are crucial for determining the ideal tools for monitoring [23]. In this sense, strategies that seek to reduce costs and shortcut the monitoring program while guaranteeing the efficiency of aquatic biodiversity biomonitoring become essential [24], including the correlations among taxonomic levels [taxonomic resolution; e.g. 25–28], between species abundance and presence-absence data of each biological group [numerical resolution; e.g. 11, 29, 30], and correlations between biological groups and their trophic subdivisions [cross-taxa; e.g. 31–33].

Taxonomic resolution represents the use of coarser taxonomic levels (e.g., genus and family) instead of more detailed identification at the species level, without significant loss of information [11, 27, 34, 35]. Thus, adopting the taxonomic resolution method brings benefits such as reduced time for identification and, consequently, costs [29, 36]. That can facilitate the development of studies with reliable information and accelerate the performance of biomonitoring programs [37]. The numerical resolution aims to replace species abundance with presence-absence data [29, 30, 38]. This method has demonstrated concordant results when using presence-absence data and the complete dataset [39]. Another advantage of using this method is the speed in the counting process, making biomonitoring faster to be performed and decreasing costs [29, 37, 40].

For aquatic groups, congruence is expected mainly for groups that respond similarly to environmental and spatial gradients [41]. In this sense, some groups such as phytoplankton and periphyton may show high congruence because of their similar environmental requirements [42] and dispersal capacity. Besides, high congruence is expected for groups linked directly through the trophic web [43], such as phytoplankton and zooplankton [44], algae (phytoplankton and epilithic algal community), herbivorous fish [45], and even macroinvertebrates and fish.

For biomonitoring purposes, detecting concordant groups and assessing one or few groups would reduce the demand for people and resources without reducing the quality of information [9, 24, 46]. However, some groups may differ in their responses to the environment, and the assessment of all these groups could help capture more detailed information on environmental variations [47]. Therefore, the multi-taxa approach [43, 47, 48] allows us to understand the concordance between groups and their response to environment and landscape variables, helping to guide optimized biomonitoring strategies.

Changes in riparian vegetation could drive changes in congruence patterns, as bioindicators from different taxonomic groups may have different tolerance thresholds to vegetation loss [e.g. 5]. For streams, the riparian forest protects the watercourse and retains sediment and contaminants from adjacent areas, acting as a buffer against the impacts of deforestation [49], in addition to maintaining the heterogeneity of aquatic habitats [50]. Many studies show a higher sensitivity of aquatic communities, not only to pollution but also to deforestation of stream riparian areas [51].

Biomonitoring is based on the assumption that bioindicators respond to environmental variations. However, this assumption is not necessarily valid, since dispersal-related processes may overcome niche processes influencing community structure and, consequently, the bioindicator responses [52]. Dispersal influences may be stronger enough to override the environmental signal captured by biological communities, making bioindicator responses unreliable [53, 54]. Thus, a crucial step in biomonitoring is identifying bioindicators less influenced by spatial processes and selecting those with stronger environmental signals [55].

Therefore, in this study, we aim to evaluate the different surrogacy methods (taxonomic, numerical, and cross-taxa) in streams, considering distinct aquatic groups. Besides, we aim to investigate whether these different levels (taxonomic and numerical) or groups have similar responses to the environmental gradient and spatial patterns. Thus, for taxonomic surrogacy (i), we expect the congruence between species and genus will be greater than between species and coarser levels (family, order, class) [11, 27]. For numerical resolution (ii), we expect the surrogacy of species abundance for presence-absence data to be highly concordant in all biological groups [26]. For the cross-taxa method (iii), we expect the cross-taxa congruence to occur between (a) phytoplankton and periphytic communities because of their similar environmental requirements, such as nutrients and luminosity [56–58]. Moreover, we expect high congruence between (b) algae (phytoplankton and periphyton) and herbivorous zooplankton [27, 44], between (c) carnivorous fish and zooplankton [59, 60], and between (d) algae (phytoplankton and periphyton) and herbivorous fish [45], due to the direct relationships established between these organisms through the food chain.

## Material and methods

### Study area

**Fig 1:**
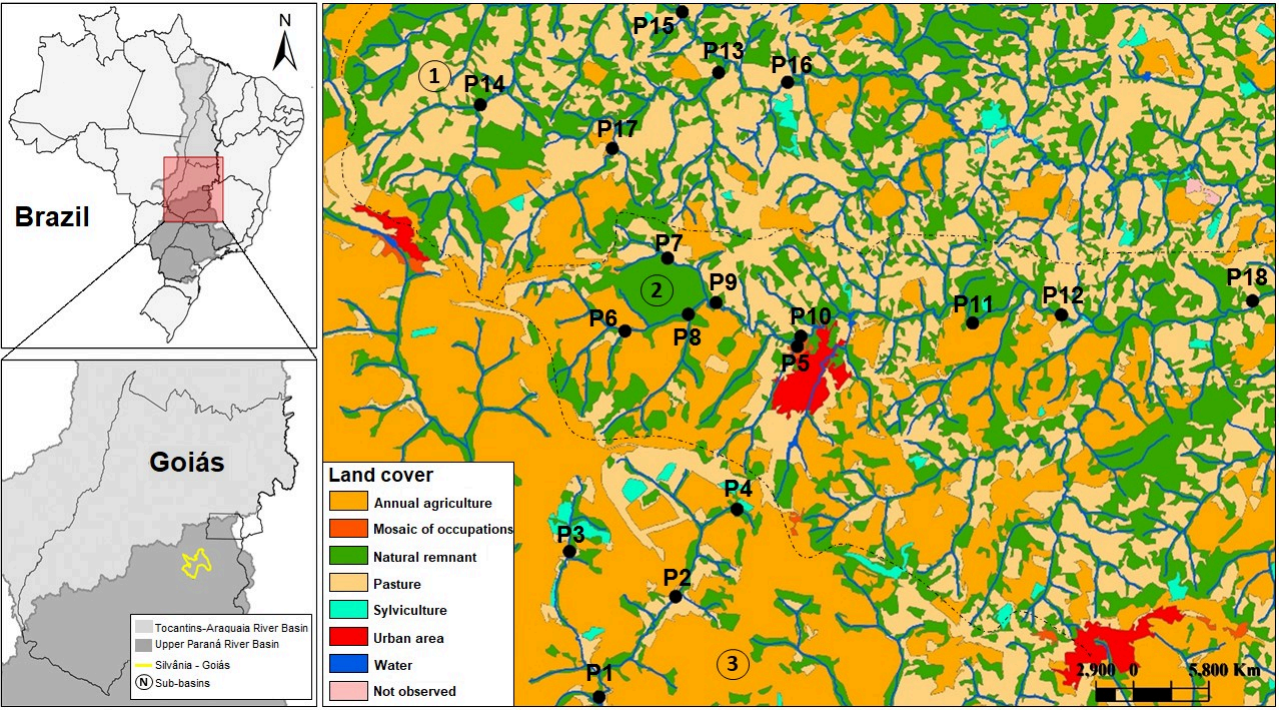
Location of the 18 sampling points in the three sub-basins, (1) Piracanjuba, (2) Ribeirão Vermelho, and (3) Rio dos Bois in the municipality of Silvânia, State of Goiás, Brazil.

The study was carried out in 18 streams of the upper Paraná River basin, including the sub-basins of Piracanjuba, Ribeirão Vermelho and Rio dos Bois. We used first- to fourth-order streams naturally shallow, narrow, and close to water sources (Fig 1). The streams are shallow, narrow, with a pH close to neutral, low concentrations of nutrients, and predominance of unconsolidated substrates. However, they show a high variation in flow and turbidity (S1 Table in S1 File). The average altitude and temperature of the region are 900 m and 26°C, respectively [61]. The region climate is classified as Aw, according to Köppen’s classification [62], being humid tropical with well-defined dry (April to September) and rainy (October to March) seasons. The region is part of the Cerrado biome with notable landscape heterogeneity, containing a preserved area with native vegetation (Silvânia National Forest—FLONA) and different land-use types (agricultural and pasture). The region surrounding the analyzed streams is constituted of a large vegetation mosaic (Fig 1), with a large and protected area (39.66%), the Silvânia National Forest (FLONA, 486.67 ha), as well as urban areas (4.45%), pastures (33.76%), agriculture (21.03%), and forestry (1.08%).

### Collection and identification of biological material

Phytoplankton, periphyton, zooplankton, and fish samplings were carried out during the same field campaign, within an 80-meter stream stretch, during the dry season (between August and September 2017). Phytoplankton was collected in areas with puddle formation on the subsurface (0.3 m) of streams, in which the water had less flow and higher light intensity. Water samples were stored in 100 ml amber glass bottles and preserved with acetic Lugol [63]. Individuals were then counted through the sedimentation technique [64], using an inverted optical microscope (Zeiss Axiovert 25) with 400x magnification. Individuals were counted in random fields until no new species were added [65]. The density was expressed in individuals per ml (ind. ml^-1^), and the individuals identified up to the lowest possible taxonomic resolution and classified taxonomically according to the system proposed by Round [66–68].

Samples of the periphytic community were collected by scraping stones, leaves, and branches with a higher visual concentration of the superficial periphytic community. Scraping was performed using a plate with a central opening of 5 cm^2^, a brush, and distilled water. After scraping and washing, the resulting content was stored in a 100 ml amber flask and fixed with acetic Lugol [63]. Periphyton samples were sub-sampled and counted following the same protocol applied for phytoplankton, using the sedimentation technique [64]. The individuals were identified and classified according to Round [66–68].

Zooplankton sampling was performed using a plankton net with 68μm mesh size, in which 300L of water was filtered at each sampling point. The water collected for filtering zooplankton was always around two meters above and below the phytoplankton collection points. Each sample was buffered in 5% formalin and concentrated to a volume of 100 ml in 250 ml white flasks. Quantitative analysis was performed using three 10 ml subsamples, counting at least 200 individuals from each large group (testate amoebae, copepods, cladocerans, and rotifers) in a Sedgewick-Rafter chamber under an optical microscope [69]. The total density was expressed in individuals per m^3^ (ind. m^3^). Zooplankton identification was performed based on specialized literature for different groups, such as testate amoebae [70–72] rotifers [28, 73, 74], cladocerans [75, 76], and copepods [77, 78]. Zooplankton was further classified according to their food preference in herbivores and carnivores, according to [79].

Fish were caught through electrofishing, using an alternating current generator (1,000 W, 300–500 V, 1–3 A) with two fishing net connect to electrical current, plus an additional net, without electricity. Electrofishing was carried out upstream, following a trajectory that explored all types of microhabitats over 80 meters [80]. After capture, the fish were anesthetized in water and Eugenol solution and then transferred to 10% formalin. After 72 hours, they were deposited in 70% alcohol to preserve them [80–83]. Individuals were identified using unpublished identification keys provided by a taxonomist (Fernando Rogério de Carvalho, Universidade Federal do Mato Grosso do Sul, Brazil). Fish were also categorized according to their trophic category into herbivores or carnivores [84]. The data used here corresponds to (Sistema de Autorização e Informação em Biodiversidade) SISBIO and (Instituto Chico Mendes de Conservação da Biodiversidade) ICMBio authorization for scientific activities number 59077–1.

### Environmental and spatial data

At each sampling point, limnological variables such as water temperature, transparency (m), pH, oxygen (O2), conductivity, dissolved oxygen (DO), turbidity, and chlorophyll-a were measured using the Eureka Manta 2 Amphibian probe. Other measurements were carried out in the laboratory, using water samples collected at the site, which were tested following Standard Methods for the Examination of Water and Wastewater [85]. The variables measured in the laboratory were biochemical oxygen demand (BOD), oxidation-reduction (redox), total solids (TS), total dissolved solids (TDS), nitrate, total nitrogen, ammonia nitrogen, total phosphorus, total organic carbon, and iron.

The habitat characterization was carried out based on measurements obtained in nine equidistant transects (10 m) within the 80-meter stretch sample. The following variables were obtained for each transect: stream width, mean depth represented by five points equidistant from one margin to another; flow, measured using the General Oceanics ® flowmeter, model 2030; riparian forest width, estimated visually up to 30 meters on each bank; stable (rocks and logs) and unstable (sand and mud) substrate composition, estimated visually as the proportion of each substrate component [86]. We then calculated the mean values and variation (standard deviation) of the environmental variables of each sampling location.

Spatial data were obtained using a distance matrix from the sampling points following the streamflow. The distances were used to create spatial vectors representing the autocorrelation among sampling points [87, 88]. From the analysis, we generated ten PCNM spatial filters (Principal coordinates of neighbor matrices) [89, 90] that were later included in our data analysis.

### Data analysis

**Fig 2:**
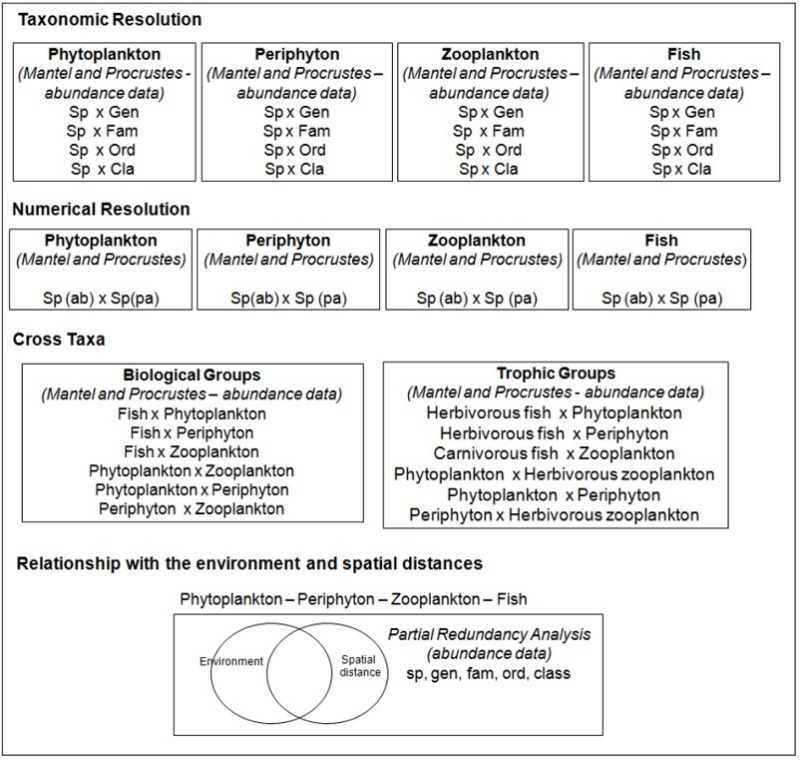
Analyses used to assess the taxonomic, numerical, and cross-taxa congruence and the effect of environmental and spatial variables for phytoplankton, periphyton, zooplankton, and fish communities in 18 streams of the sub-basins Piracanjuba, Ribeirão Vermelho, and Rio dos Bois, in the municipality of Silvânia, State of Goiás, Brazil. sp = species; gen = genus; fam = family, ord = order; cla = class; ab = abundance; pa = presence or absence.

Mantel and Procrustes analyses [91] were used to assess the congruence among taxonomic levels (i.e., species with higher levels such as genus, family, order, and class), numerical data types (species abundance and occurrence–S1 Table in S1 File), biological groups (phytoplankton, periphyton, zooplankton, and fish), and trophic levels (herbivorous fish, carnivorous fish, and herbivorous zooplankton). Both tests assess the correlation between multivariate datasets [92]. Mantel is a correlation test between two pairwise matrices [93] and Procrustes is a correlation test between ordination analysis axes [94]. The result interpretation in both tests is similar, in which statistic r varies between 0 (no congruence) and 1 (perfect congruence). We consider that correlations (Mantel and Procrustes) higher than 0.7 [24] suggest a strong concordance among the taxonomic levels, numerical data, biological groups, and trophic categories evaluated. For all correlations, the significance of r values was tested using the Monte Carlo method with 10,000 random permutations. All analyses were performed using the vegan package [95] in the R software [96]. A summary of the analyses performed in this study is present in Fig 2.

#### Taxonomic resolution

The abundance (for fish) and density (for zooplankton, phytoplankton, and periphyton) data were log-transformed (log X + 1) to remove the effect of high abundant species [97]. The abundance matrices for the different taxonomic levels (species, genus, family, order, and class) were converted into Bray-Curtis distance matrices and correlated using the Mantel’s test. Subsequently, the Bray-Curtis distance matrices for each taxonomic level were submitted individually to Non-Metric Multidimensional Scaling analysis (NMDS). The literature suggests using the number of axes with the best fits [98]; thus, we selected the first two NMDS axes generated for each taxonomic level and correlated them using the Procrustes analysis.

#### Numerical resolution

In this step, two types of datasets were used for each biological group individually (phytoplankton, periphyton, zooplankton, and fish). They are matrices of species composition based on abundance and occurrence (presence and absence). The abundance data were log-transformed (log X + 1) and converted to Bray-Curtis distance matrices, while the occurrence data were converted to Jaccard distance matrices. Subsequently, these matrices were correlated among them for each biological level individually using Mantel’s test. They were then used to perform the NMDS, whose first two axes generated for each matrix type were correlated using the Procrustes analysis.

#### Cross-taxa congruence

We used matrices of species composition based on abundance data for each biological group and its trophic subdivisions to assess the surrogate groups. To fulfill our hypotheses, the fish group was categorized into total fish (considering the abundance of all sampled individuals), herbivorous fish, and carnivorous fish. Similarly, the zooplankton group was categorized into total zooplankton (considering the abundance of all sampled individuals) and herbivorous zooplankton (considering the abundance of herbivorous individuals only). The phytoplankton and periphyton groups were not categorized into subgroups. All data were log-transformed (log X + 1) and converted into Bray-Curtis distance matrices per biological or trophic groups. These matrices were correlated through the Mantel’s test and used to perform the NMDS, whose first two axes, generated for a given biological group or its trophic subdivision, were correlated with the axes generated for the others, contemplating all possible combinations.

#### Relationship with the environment and spatial patterns

A partial redundancy analysis (pRDA) was used to evaluate the influence of environmental variables and spatial patterns on the abundance of species, genera, families, orders, and classes. We considered all the biological groups previously evaluated because they show responses similar to the environmental gradient or the dispersal patterns throughout the streams.

For pRDA, different environmental variables were selected according to each group analyzed (fish, phytoplankton, periphyton, and zooplankton), based on the ecological relationships of groups with the environment already observed in the literature. The Variance Inflation Factors (VIF) was calculated to remove collinear variables. We consider variables with VIF > 0.5 to be collinear. The VIF was calculated using the "vifcor" function from the usdm package [99]. The environmental variables (except pH) were log-transformed (log X + 1) to obtain a normal distribution or closer to normal for variables.

We used the forward selection function of the adespatial package [100] to select the explanatory variables. In forward selection, were utilized the different environmental variables selected previously according to VIF analysis and all PCNM filters previously generated. The selection of environmental and spatial predictors was performed individually using two stopping criteria, (1) the pre-selected significance level (P < 0.05) and (2) the global model statistic, where the predictors explain the significance in which the variation in community composition was maintained (P < 0.05) [101]. The forward selection was used individually for the taxonomic categories (species, genus, family, order, and class) so that the predictors varied depending on the response variable used.

A variation partitioning approach with adjusted R2-based redundancy analysis (RDA) was carried out to determine the relative importance of local environmental and spatial components [90, 91]. Subsequently, different pRDAs were performed based on the selected predictive, spatial, and environmental variables [91, 102]. The significance of each variation component was tested using the anova function, while RDA and pRDA were performed using the rda and varpart functions, respectively, of the vegan package [90]. All statistical analyses were performed in the R software [96], considering a 5% significance level (P values < 0.05).

## Results

**Fig 3:**
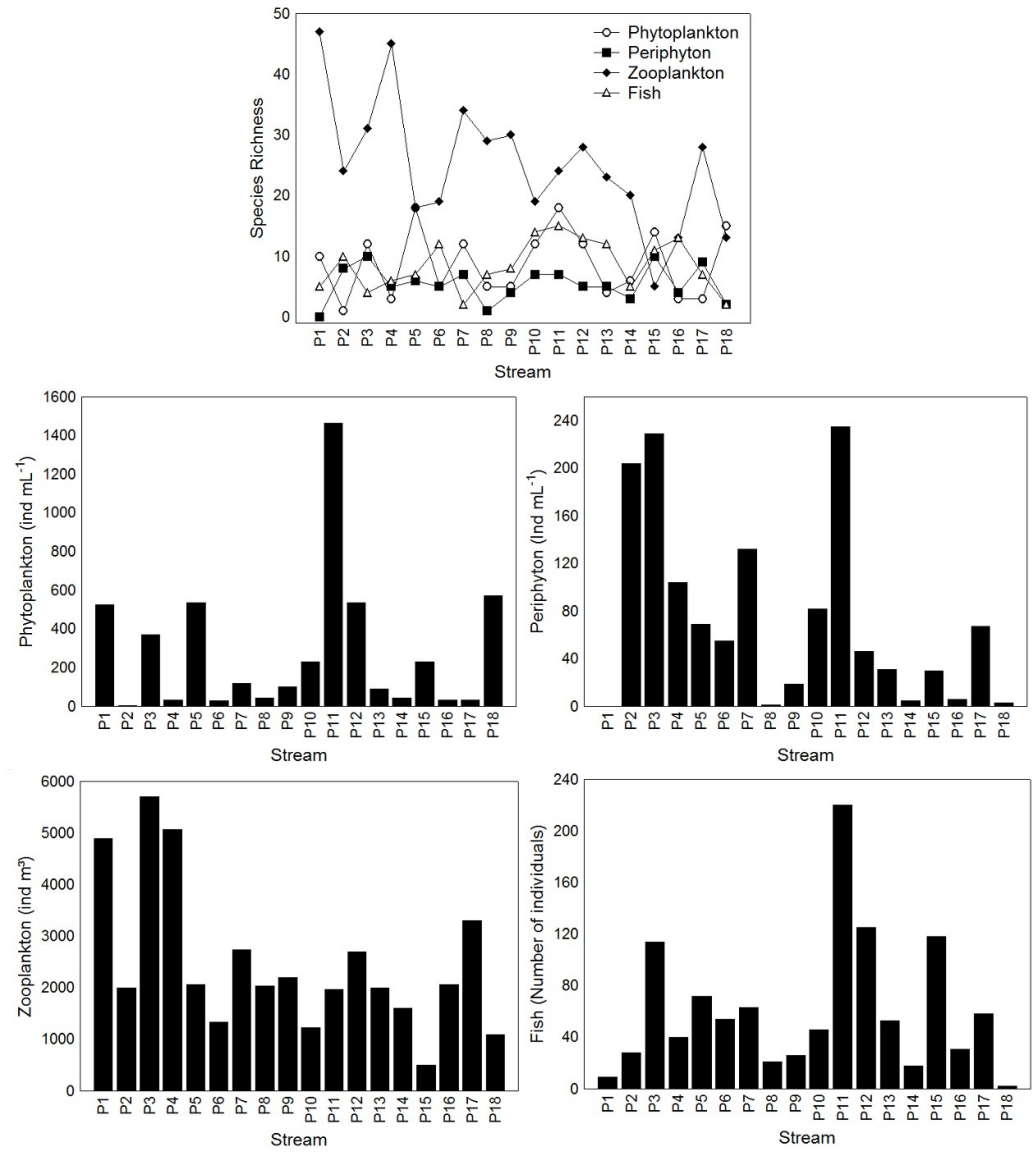
Species richness and total abundance or density observed for phytoplankton, periphyton, zooplankton, and fish in 18 streams of the sub-basins Piracanjuba, Ribeirão Vermelho, and Rio dos Bois in the municipality of Silvânia, State of Goiás, Brazil. ind = individuals.

We identified 33 fish species, distributed into 26 genera, 18 families, and six orders, totalizing 1098 individuals. The lowest species richness and abundance was observed in point 18 and the highest in point 11 (Fig 3). The most abundant species were *Bryconamericus turiuba*, *Piabina argentea*, and *Poecilia reticulata*, corresponding to 40% of total fish abundance (S2 Table in S1 File). For phytoplankton, we identified 68 species, 39 genera, 28 families, 19 orders, and ten classes. The lowest species richness and total density was observed in point 2 and the highest in point 11 (Fig 2). The species with the highest density of individuals were *Monoraphidium griffithii*, *Navicula* sp., and *Chrococcus minimus* (S2 Table in S1 File). The periphytic community had 41 species, 29 genera, 25 families, 19 orders, and eight classes. The lowest species richness was observed in point 8 and the highest in points 3 and 15 (Fig 2). The lowest total density was observed in point 8 and the highest in point 11 (Fig 2). In total, we observed 1318 periphyton individuals, of which the most abundant species were *Sellaphora* sp., *Navicula* sp., *Pseudonabaena* sp., and *Gloeocapsopsis* sp., respectively (S2 Table in S1 File). For zooplankton, we identified 88 species, 33 genera, 23 families, six orders, and six classes. The lowest species richness was observed at point 15 and the highest at point 1 (Fig 2). The lowest total density was observed at point 15 and the highest at point 3 (Fig 2). The most abundant species were *Moina micrura*, *Bosminopsis deitersi*, and *Thermocyclops minutus* (S2 Table in S1 File).

In general, the Mantel and Procrustes analyses (Table 1 and S1–S4 Figs) showed concordant results. The comparison by taxonomic resolution showed that, the species level is congruent with the genus level for all groups (phytoplankton, periphyton, zooplankton, and fish). We found values higher than 0.9 (for Mantel and Procrustes) for the fish group. This congruence indicates that species and genus levels have similarity patterns over the sampling units. This result was slightly less consistent for the phytoplankton group since only Mantel’s test suggests congruence of species and genus. Besides, for all groups, the congruence values decreased with the increase in the taxonomic resolution level.

**Table 1:** Mantel’s and Procrustes tests using abundance (ab) and presence-absence (pa) matrices for taxonomic groups of fish, phytoplankton, periphyton, and zooplankton.

	Mantel		Procrustes	
	r	P	r	P
**Fish taxonomic resolutions (ab)**				
Species vs. Genus	**0.92**	<0.001	**0.93**	<0.001
Species vs. Family	**0.83**	<0.001	**0.88**	<0.001
Species vs. Order	**0.78**	<0.001	**0.78**	<0.001
Species vs. Class	0.39	0.01	0.405	0.08
**Phytoplankton taxonomic resolutions (ab)**				
Species vs. Genus	**0.81**	<0.001	0.61	<0.001
Species vs. Family	**0.75**	<0.001	0.58	0.001
Species vs. Order	**0.76**	<0.001	0.66	<0.001
Species vs. Class	0.69	<0.001	0.54	0.01
**Periphyton taxonomic resolutions (ab)**				
Species vs. Genus	**0.88**	<0.001	**0.782**	<0.001
Species vs. Family	**0.88**	<0.001	**0.787**	<0.001
Species vs. Order	**0.87**	<0.001	**0.83**	<0.001
Species vs. Class	0.61	<0.001	0.53	0.01
**Zooplankton taxonomic resolutions (ab)**				
Species vs. Genus	**0.76**	<0.001	**0.705**	<0.001
Species vs. Family	**0.72**	<0.001	0.63	<0.001
Species vs. Order	0.54	<0.001	0.56	0.001
Species vs. Class	0.51	<0.001	0.52	0.005
**Numerical resolutions**				
Fish species (ab) vs. Fish species (pa)	**0.89**	<0.001	**0.87**	<0.001
Phytoplankton species (ab) vs. Phytoplankton species (pa)	**0.97**	<0.001	**0.97**	<0.001
Periphyton species (ab) vs. Periphyton species (pa)	**0.89**	<0.001	**0.94**	<0.001
Zooplankton species (ab) vs. Zooplankton species (pa)	**0.98**	<0.001	**0.94**	<0.001
**Biological substitute group—Total**				
Fish (ab) vs. Phytoplankton (ab)	0.04	0.34	0.21	0.76
Fish (ab) vs. Periphyton (ab)	0.22	0.05	0.39	0.16
Fish (ab) vs. Zooplankton (ab)	-0.006	0.49	0.32	0.36
Phytoplankton (ab) vs. Zooplankton (ab)	-0.02	0.53	0.25	0.64
Phytoplankton (ab) vs. Periphyton (ab)	0.13	0.14	0.45	0.07
Periphyton (ab) vs. Zooplankton (ab)	-0.13	0.83	0.25	0.62
**Biological surrogate group—Trophic**				
Herbivorous fish (ab) vs. Phytoplankton (ab)	-0.02	0.52	0.23	0.69
Herbivorous fish (ab) vs. Periphyton (ab)	0.22	0.07	0.31	0.38
Carnivorous fish (ab) vs. Zooplankton (ab)	-0.01	0.52	0.209	0.75
Phytoplankton (ab) vs. Herbivorous zooplankton (ab)	-0.05	0.69	0.35	0.27
Phytoplankton (ab) vs. Periphyton (ab)	0.13	0.14	0.45	0.07
Periphyton (ab) vs. Herbivorous zooplankton (ab)	0.08	0.21	0.36	0.22

Significant r values above 0.7 were highlighted.

In the numerical resolution (Table 1), all biological groups showed a correlation above 0.8, indicating that the patterns of sampling units using abundance data were similar to the ordination of sampling units using presence-absence data. Thus, it is possible to use presence-absence data as a surrogate of species abundance for all groups studied. However, we found no significant correlations between biological and trophic groups (Table 1).

Fish and zooplankton were significantly correlated with the environmental variables, but not with spatial patterns (Table 2). For fish, conductivity and ORP were significant for species level and width was significant for genus level; while for zooplankton, pH and dissolved oxygen were the most important variable for most taxonomic resolutions. For phytoplankton, none of the environmental variables were significant for species, genus, family, order, and class (Table 2), but spatial patterns explained 3% and 17% of the composition identified at the genus and order level, respectively. For periphyton, spatial patterns significantly explained all levels of taxonomic identification (except species). In this group, fine scale (6, and 10) and large-scale (1 and 3) spatial patterns were significant. Considering the taxonomic resolution, the class category had the highest predictability, with 55% of its variability explained by the spatial variables.

**Table 2:** Relationship analysis between fish, phytoplankton, periphyton, and zooplankton with environmental and spatial variables for the region of Silvânia, Goiás, Brazil, in 2017.

Group	N. Category	Environment	Spatial filters	R^2^ adjusted [Environment]	R^2^ adjusted [Spatial]	P [Env.]	P [S.V.]
**Fish species**	33	Conductivity, ORP	-	0.08	-	0.01*	-
**Fish genus**	26	Width	-	0.05	-	0.01*	-
**Fish family**	18	-	-	-	-	-	-
**Fish order**	6	-	-	-	-	-	-
**Phytoplankton species**	68	-	-	-	-	-	-
**Phytoplankton genus**	40	-	1	-	0.03	-	0.05
**Phytoplankton family**	28	-	-	-	-	-	-
**Phytoplankton order**	19	-	1,2,3	-	0.17	-	0.002*
**Phytoplankton class**	10	-	-	-	-	-	-
**Periphyton species**	42	STD	1,3	-0.01	0.03	0.61	0.17
**Periphyton genus**	29	-	1,6	-	0.16	-	0.001*
**Periphyton family**	25	-	1,6	-	0.16	-	0.003*
**Periphyton order**	19	-	1.6	-	0.22	-	0.001*
**Periphyton class**	8	-	1,3,6,10	-	0.55	-	0.002*
**Zooplankton species**	88	-	-	-	-	-	-
**Zooplankton genus**	33	pH, DO	-	0.13	-	0.003*	-
**Zooplankton family**	23	pH, DO	-	0.16	-	0.001*	-
**Zooplankton order**	6	pH	-	0.17	-	0.002*	-
**Zooplankton class**	6	pH	-	0.17	-	0.006*	-

The code “-”indicate that no variable was selected in the global model by the forward selection function (see details in Methods), and it was not possible to proceed with the analysis. Significant values (*P<0.05) indicate what environmental (ENV.) or spatial variables (S.V.) were associated with the variation in each species group. N indicates the number of different organisms in a given level of taxonomic identification. No analyses were performed for the class category since all fish sampled belong to the class Actinopterygii.

## Discussion

In this study, we found a high congruence among the taxonomic levels of all the biological groups evaluated, as well as between the species occurrence and abundance data. These results indicate that all biological groups showed a strong congruence between species identification and higher taxonomic levels and between presence-absence and abundance data, corroborating predictions i and ii. However, we did not find cross-taxa congruence between biological or trophic groups, contradicting the prediction iii. Therefore, replacing one group with another would result in loss of significant information since the groups showed complementary responses, capturing different aspects of the environmental variation of streams. In this sense, the simplification in the taxonomic levels or numerical resolution may result in cutting expenses of multiple collection campaigns, specialized taxonomists, and labor-intensive and time-consuming processes, without interfering with the quality of the process [26, 103–105]. Thus, future studies can estimate how much can be saved by applying simplification protocols in this region [e.g. 106].

### Taxonomic resolution

Taxonomic resolution analysis with Mantel’s test showed a high correlation (r > 0.7) for all groups (fish, phytoplankton, periphyton, and zooplankton), indicating that species-level classification can be replaced by genus and even family, without significant loss of biological information. Thus, higher taxonomic levels (genus, family, and order) also represent the diversity of local species and may act as surrogates for environmental assessments [35]. These results corroborate several studies proposed for different aquatic groups, such as phytoplankton [13, 25–27], periphyton [16], macroinvertebrates [107], and fish [16, 26, 108, 109], indicating that the taxonomic resolution can be applied even for assessment of environmental impact [110].

Fish and periphyton groups had the highest correlation values (Mantel’s r > 0.9) for the genus level. For the periphyton, this high correlation remained for family (Mantel’s r > 0.9) and order (Mantel’s r > 0.8) levels. These results corroborate studies involving periphyton and fish [24, 20]. They show a high level of community concordance in small geographical areas, representing a community structure [24], a fact corroborated in this study for fish and periphyton. That also indicates that groups with more ability to choose or stay in habitats (e.g. fish and periphyton) showed higher correlation values than those carried more easily by the water flow (e.g. phytoplankton and zooplankton). These results can be explained by the fact that most fish species captured were specialists with efficient dispersal abilities; or because the periphyton species remain attached to a substrate, controlling its dispersion and spatial variation.

In general, the use of coarser taxonomic resolutions for stream environments is also a reliable and robust option for rapid bioassessment studies that require fewer resources [111]. They are used principally for groups with multiple families and genera (e.g., periphyton) and robust taxonomic congruence data [16]. Moreover, taxonomic identification at the species level requires specialists for different groups, which is not always available and can take a long time [50], especially in regions with high diversity [16, 112]. For this reason, the use of coarser taxonomic resolutions is a viable possibility for rapid bioassessment. However, it is necessary to emphasize that this surrogacy must be done with caution, because many species may still be unknown, mainly in regions of high biodiversity [16], if possible, the biomonitoring should be done with multiple taxonomic groups, and this approach is not efficient to propose, select, and monitor areas for conservation purposes [113]. In these regions, other factors must be taken into account, such as functional similarity [113, 114].

### Numerical resolution

The numerical resolution aims to use presence-absence data as a surrogate for species abundance, which can speed up the assessment of communities, and consequently, the biomonitoring. Our results showed the possibility of surrogacy for all groups (fish, phytoplankton, periphyton, and zooplankton), with a high correlation (r > 0.8).

Moreover, the use of presence-absence data can simplify and reduce time and effort in the biomonitoring of aquatic ecosystems since all groups mentioned in this study have been used as bioindicators of these ecosystems [115]. Presence-absence data remove abundance differences, and as a consequence, reduce the effect of dominant species regarding the ordination [39, 116]. Furthermore, rare species can be as significant as common species in studies of species-environment relationships [117, 118]. Abundance data, on the other hand, demand more time for individuals counting. However, when necessary, these data allow a more distinct assessment of the most subtle ecological patterns in the community structure [39, 119] and niche selection [120], which is not easily observed with presence-absence data.

### Surrogate groups

The concordance between biological groups and trophic divisions was not significant for any studied biological groups. Therefore, the ordination patterns recorded for one group did not correspond to other groups, even those with similar environmental requirements (e.g., phytoplankton and periphyton) or related in the food chain (e.g., periphyton and herbivorous fish). Thus, no biological or trophic group can be used as a surrogate for another in the region evaluated. This pattern corroborates other studies [26, 121] and highlights the particular importance of each biological group in environmental monitoring and biodiversity assessments [60, 122] since different groups respond to different scales of spatial and temporal impacts. For example, some groups may respond to limnological variables and others to hydrological variables, such as the presence or absence of riparian forest, while others are responding to changes over time [39, 123, 124].

The lack of cross-taxa congruence may occur due to different responses of the groups to the distinct spatial scale variations in their core area [125, 126], as well as the life history of species [127, 128]. Despite the small number of streams studied, we covered a large part of the conservation unit area, contemplating a reasonable environmental variation in the region. Furthermore, the biodiversity sampled in the region is in accordance with the biodiversity found in other studies developed in the Brazilian Cerrado streams [e.g. 26, 129]. Thus, the small spatial scale and the significant anthropogenic influence on the study area may be interfering with the congruence among different groups.

Therefore, in this region, the use of cross-taxa may not be the most suitable for stream biomonitoring and small-scale assessments. Heino [24] also shows that the fish community assessment may depend on multiple environmental variables, according to their different environmental requirements. Besides, Barbosa *et al*. [26] indicate that for monitoring purposes, different groups must be evaluated in different ways, covering different strata of local trophic levels since these do not demonstrate a possibility of surrogacy among distinct communities. In fact, while assessing the fish’s responses to environmental conditions, they were more associated with structural variables in streams, such as width and flow. The phytoplankton and periphyton was not directly associated with any of the evaluated environmental variables, while zooplankton was associated with physicochemical variables, such as dissolved oxygen and pH. These results indicate the need for complementarity of groups on aquatic biodiversity biomonitoring, mainly between fish and phytoplankton, fish and periphyton, and fish and zooplankton, since they respond to different environmental characteristics.

The trophic groups did not corroborate the expected responses. In this case, the ecological functions may be more strongly connected with changes in habitats and environmental interference on the ichthyic stream assemblage [5, 130]. Thus, by grouping the fishes into broad categories, we may have lost the diet variation in quantitative terms that may be more connected to the impact on phytoplankton and zooplankton groups. These environments are small and strongly impacted by the maintenance or not of riparian areas, which can directly reflect the community’s dietary conditions, yet not considered in this study. Other parameters, such as biomass of bacteria and fungi in the periphery (important for zooplanktons) and entry of allochthonous material (important for fishes), could also influence the responses.

### Relationship with the environment and spatial patterns

The importance of environmental gradients to predict responses for the species level are recurrent in many studies [e.g. zooplankton; 131]. This taxonomic level shows specific criteria for survival, which may be linked to the heterogeneity of aquatic ecosystems and environmental variables, which directly influence species composition [132, 133]. However, we emphasize that our results demonstrate correlation with environmental variables for species and genus of fish; genus, family, order and class of zooplankton; higher importance of spatial patterns for the most taxonomic resolutions of periphyton, and absence of environmental and spatial explanation for most taxonomic levels of phytoplankton.

Furthermore, the dynamics and structure of communities are not defined only by environmental variables. Instead, other factors such as spatial pattern [134], climate [135], biological interactions, and dispersal limitation [136] may play important roles in communities. However, our results showed significant relationships among the taxonomic levels and that these respond similarly to the environmental variables or spatial patterns. That allows us to use surrogate taxonomic categories within the same group, for example, by replacing species level with a higher taxonomic category, which would facilitate biomonitoring. However, surrogacy among groups is not possible because they are not correlated, probably because they are influenced by different environmental variables and spatial patterns.

## Conclusion

Given the ecological importance of stream communities, biomonitoring is fundamental to understand the anthropogenic effect and plan efficient management for ecosystem conservation and restoration. Our results showed high congruence, especially for taxonomic and numerical resolutions. Thus, in general, the four groups evaluated (phytoplankton, periphyton, zooplankton, and fish) have a high biomonitoring potential on local scales. Moreover, the biological groups showed complementary responses to environmental gradients. Therefore, our results support the multi-taxa approach, using less detailed taxonomic and numerical resolutions in biomonitoring programs. The possibility of simplification in the sampling protocols found in our study can reduce costs for monitoring in this region. In this sense, future studies can focus on estimating how much this simplification can bring to biomonitoring.

## Supporting information

S1 Fig First and second axes obtained in the Non-Metric Multidimensional Scaling analysis (NMDS) for phytoplankton. (TIF)

S2 Fig First and second axes obtained in the Non-Metric Multidimensional Scaling analysis (NMDS) for periphyton. (TIF)

S3 Fig First and second axes obtained in the Non-Metric Multidimensional Scaling analysis (NMDS) for zooplankton. (TIF)

S4 Fig First and second axes obtained in the Non-Metric Multidimensional Scaling analysis (NMDS) for fish. (TIF)

S1 File Table of mean and standard deviation for the environmental variables measured in the 18 streams in the Brazilian Cerrado and scripts analysis for R Program. (DOC)
