# Supplementary figures and images for: Shortcuts for biomonitoring programs of stream ecosystems: Evaluating the taxonomic, numeric, and cross-taxa congruence in phytoplankton, periphyton, zooplankton, and fish assemblages
Source: PLoS One. 2021 Oct 14;16(10):e0258342. doi: 10.1371/journal.pone.0258342 (PMC8516258; doi:10.1371/journal.pone.0258342)

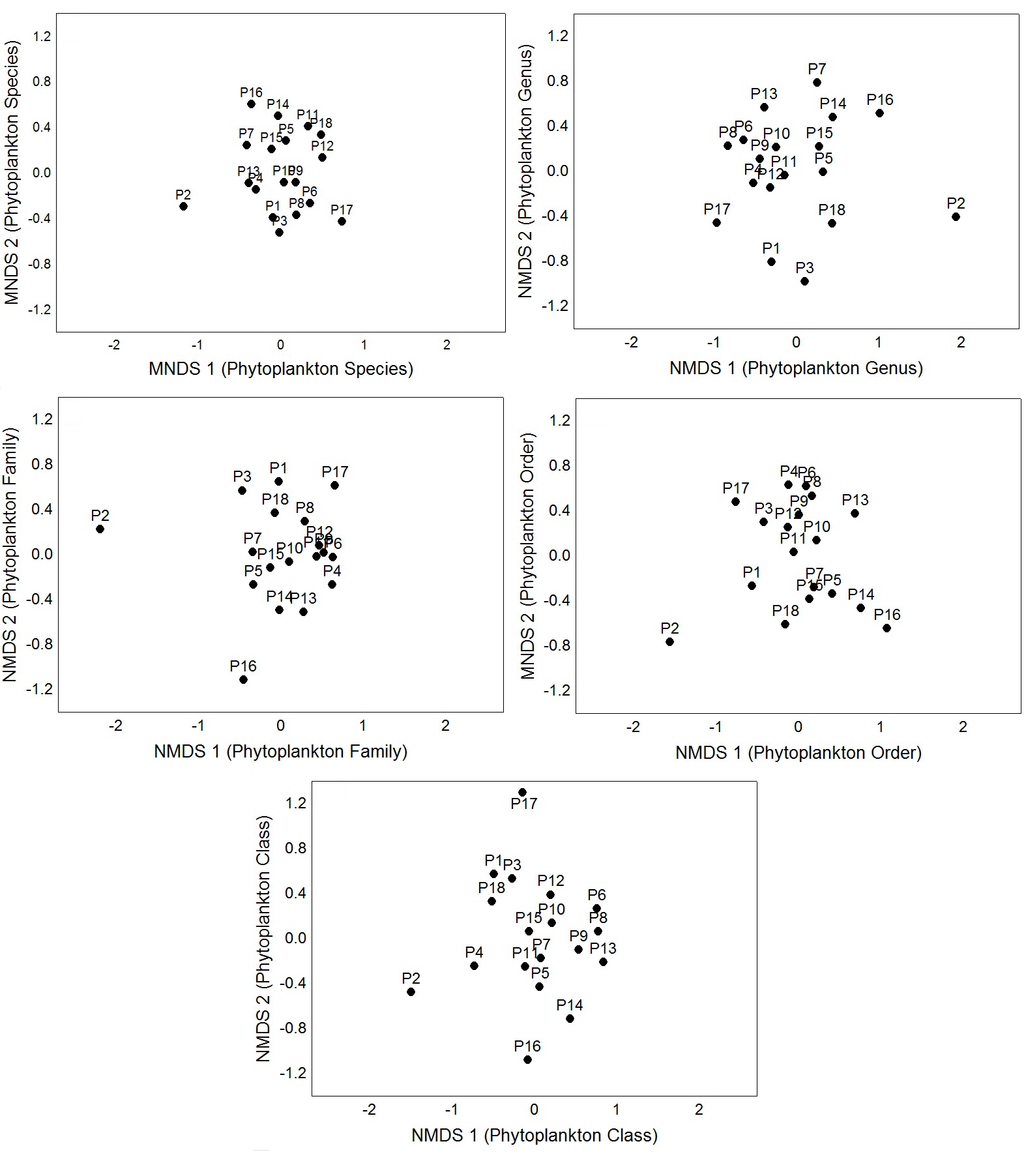

Supplement: S1 Fig — (TIF) [file pone.0258342.s001.tif]

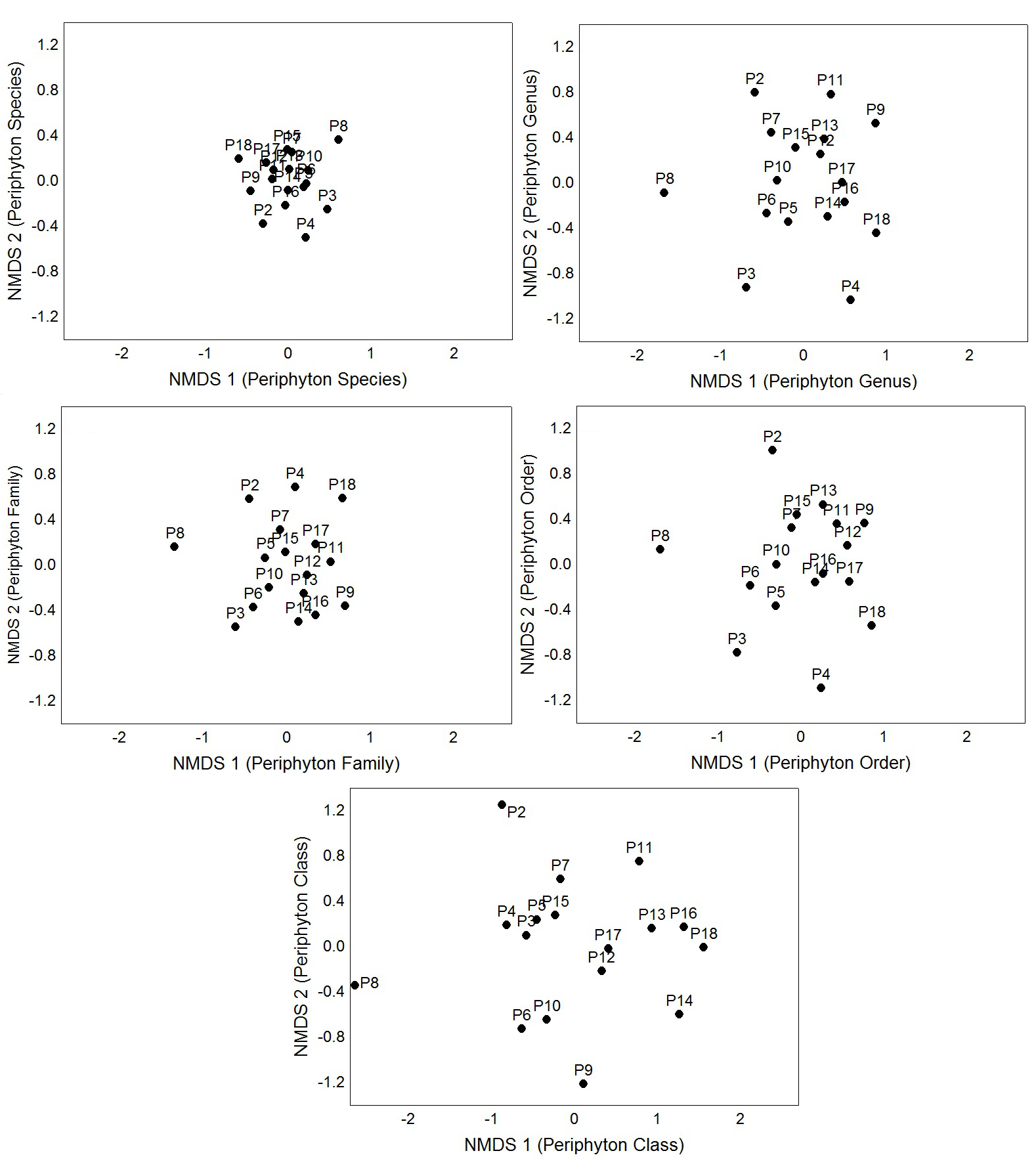

Supplement: S2 Fig — (TIF) [file pone.0258342.s002.tif]

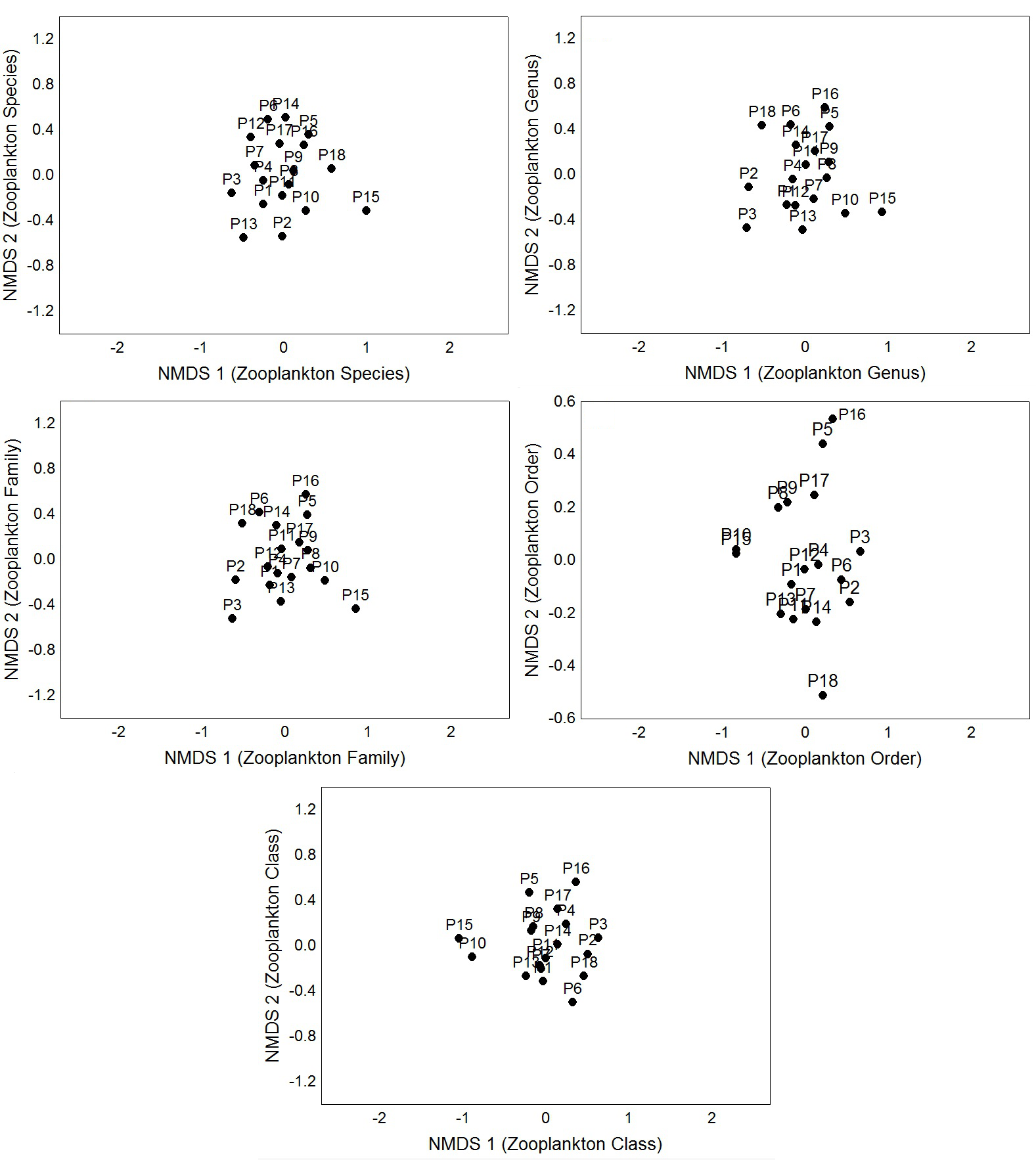

Supplement: S3 Fig — (TIF) [file pone.0258342.s003.tif]

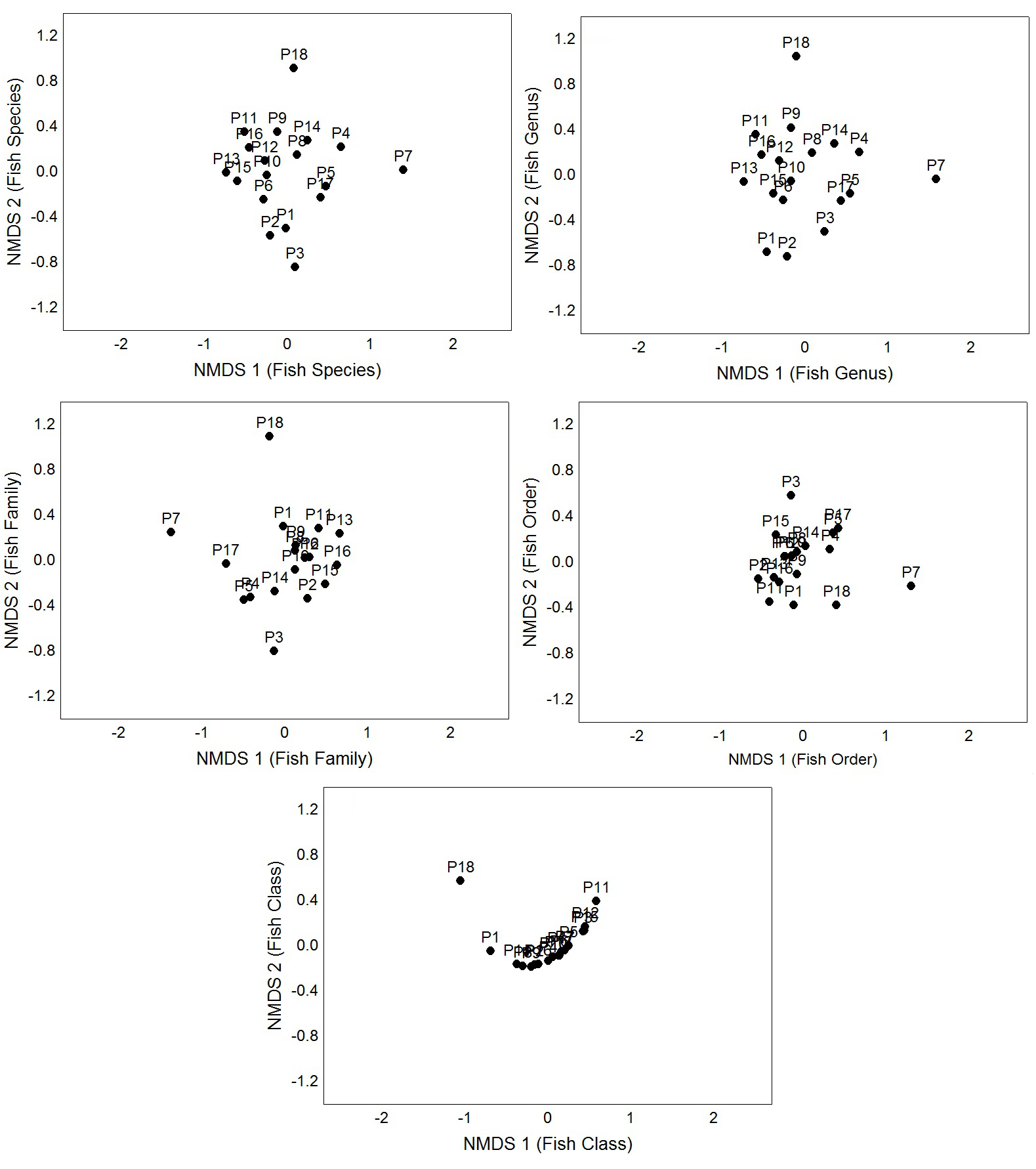

Supplement: S4 Fig — (TIF) [file pone.0258342.s004.tif]
