# Supplementary material for: Shortcuts for biomonitoring programs of stream ecosystems: Evaluating the taxonomic, numeric, and cross-taxa congruence in phytoplankton, periphyton, zooplankton, and fish assemblages
Source: PLoS One. 2021 Oct 14;16(10):e0258342. doi: 10.1371/journal.pone.0258342 (PMC8516258; doi:10.1371/journal.pone.0258342)
Supplement: S1 File — (DOC) [file pone.0258342.s005.DOC]

**Shortcuts for biomonitoring programs of stream ecosystems: evaluating the taxonomic, numeric, and cross-taxa congruence in phytoplankton, periphyton, zooplankton, and fish assemblages**

**Short title: Shortcuts for biomonitoring programs of stream ecosystems**

**Ruan Carlos Pires Faquim¹***^¶^**, Karine Borges Machado^2^**^&^**, Fabrício Barreto Teresa¹**^&^**, Pedro Henrique Francisco de Oliveira^2^**^¶^**, Gustavo Fernandes Granjeiro^3^**^¶^**, Ludgero Cardoso Galli Vieira^3^**^&^**, João Carlos Nabout¹**^&^

1. Câmpus Anápolis de Ciências Exatas e Tecnológicas - Henrique Santillo, Universidade Estadual de Goiás, Anápolis, Goiás, Brazil
2. Departamento de Ecologia, Instituto de Ciências Biológicas, Universidade Federal de Goiás, Goiânia, Goiás, Brazil
3. Faculdade de Planaltina, Universidade de Brasília, Planaltina, Distrito Federal, Brazil

* Corresponding author

E-mail: ruan_faquim@hotmail.com

**SUPPORTING INFORMATION 1**

|  | **Mean** | **Stand.**  **Dev.** | **P1** | **P2** | **P3** | **P4** | **P5** | **P6** | **P7** | **P8** | **P9** | **P10** | **P11** | **P12** | **P13** | **P14** | **P15** | **P16** | **P17** | **P18** |
| --- | --- | --- | --- | --- | --- | --- | --- | --- | --- | --- | --- | --- | --- | --- | --- | --- | --- | --- | --- | --- |
| **Temperature (°C)** | *18.63* | *1.68* | 17.7 | 19.92 | 17.63 | 20.59 | 19.12 | 19.38 | 19.35 | 19.76 | 16.55 | 19.19 | 16.45 | 17.4 | 18.5 | 16.86 | 21.75 | 21.12 | 18.33 | 15.8 |
| **Transparency (%)** | *0.80* | *0.31* | 1 | 1 | 1 | 1 | 0.1 | 1 | 1 | 1 | 1 | 1 | 1 | 0.95 | 1 | 1 | 0.5 | 0 | 1 | 1 |
| **pH** | *6.59* | *0.44* | 6.05 | 5.62 | 6 | 6.47 | 6.84 | 6.56 | 7.25 | 7.43 | 6.84 | 6.93 | 6.4 | 6.68 | 6.63 | 6.43 | 7.03 | 6.7 | 6.54 | 6.47 |
| **Oxygen*** | *7.60* | *0.79* | 7.94 | 7.6 | 5.94 | 6.88 | 7.33 | 8.08 | 5.48 | 7.86 | 8.57 | 7.79 | 7.51 | 8 | 7.76 | 8.41 | 7.87 | 7.73 | 8.26 | 7.8 |
| **Conductivity#** | *41.18* | *23.20* | 16.4 | 8.01 | 15.8 | 9.5 | 82 | 26.6 | 64.03 | 24 | 28.7 | 40.1 | 75.07 | 76.5 | 46.7 | 43.94 | 43.7 | 39.4 | 39.6 | 62.27 |
| **DO*** | *7.56* | *0.79* | 7.9 | 7.6 | 5.9 | 6.9 | 7.3 | 8.0 | 5.4 | 7.8 | 8.5 | 7.7 | 7.5 | 8.0 | 7.7 | 8.4 | 7.8 | 7.7 | 8.2 | 7.8 |
| **Turbidity (NTU)** | *69.80* | *165.78* | 5.47 | 4.63 | 10.58 | 4.8 | 118.9 | 15.49 | 10.1 | 6.15 | 4.95 | 14.56 | 6.09 | 5.75 | 4.62 | 4.78 | 16.52 | 604.1 | 3.01 | 4.18 |
| **Chl-a*** | *1.35* | *1.58* | 2.81 | 0.17 | 1.25 | 0.17 | 2.54 | 0.34 | 1.25 | 0.3 | 0.38 | 0.37 | 6.56 | 2.11 | 0.49 | 0.237 | 0.507 | 2.65 | 1.29 | 0.95 |
| **BOD*** | *1.74* | *1.68* | 0.4 | 4.50 | 4.20 | 2.10 | 0.90 | 1.70 | 4.60 | 5.00 | 0.34 | 0.90 | 0.10 | 0.30 | 0.90 | 0.10 | 1.30 | 2.40 | 1.00 | 0.60 |
| **ORP (mV)** | *268.93* | *73.20* | 358.8 | 256.03 | 330 | 185.6 | 304.8 | 216.2 | 108.05 | 194.4 | 311.1 | 215.4 | 339.5 | 325.3 | 325 | 186.2 | 226.7 | 294 | 289.8 | 374 |
| **TDS*** | *20.59* | *11.64* | 7.72 | 4.00 | 7.90 | 4.75 | 41.00 | 13.30 | 32.01 | 11.00 | 14.30 | 21.20 | 37.50 | 38.25 | 23.35 | 21.97 | 21.85 | 19.70 | 19.80 | 31.13 |
| **T. Solids*** | *55.44* | *54.39* | 15.00 | 10.00 | 32.00 | 16.00 | 89.00 | 31.00 | 48.00 | 117.00 | 33.00 | 34.00 | 52.00 | 47.00 | 35.00 | 42.00 | 51.00 | 248.00 | 42.00 | 56.00 |
| **Nitrate*** | *0.66* | *0.742* | 0.30 | 0.30 | 0.30 | 0.30 | 1.20 | 0.30 | 0.80 | 0.30 | 0.30 | 0.40 | 1.20 | 0.50 | 0.40 | 0.70 | 0.40 | 3.40 | 0.30 | 0.60 |
| **T. Nitrogen*** | *0.89* | *1.12* | 0.342 | 0.344 | 0.346 | 0.353 | 1.761 | 0.114 | 1.555 | 0.225 | 0.344 | 0.525 | 1.564 | 0.594 | 0.563 | 0.893 | 0.544 | 4.968 | 0.375 | 0.744 |
| **A. Nitrogen*** | *0.24* | *0.37* | 0.04 | 0.04 | 0.24 | 0.05 | 0.54 | 0.03 | 0.75 | 0.03 | 0.04 | 0.12 | 0.36 | 0.08 | 0.16 | 0.09 | 0.11 | 1.55 | 0.07 | 0.14 |
| **T. Phosphorus*** | *0.020* | *0.03* | 0.003 | 0.003 | 0.011 | 0.003 | 0.020 | 0.003 | 0.126 | 0.003 | 0.003 | 0.003 | 0.116 | 0.051 | 0.003 | 0.003 | 0.003 | 0.016 | 0.003 | 0.003 |
| **T. Org. Carbon*** | *6.19* | *2.72* | 7.3 | 3.9 | 7.0 | 4.5 | 4.8 | 4.6 | 6.8 | 7.4 | 3.6 | 3.9 | 14.7 | 4.9 | 6.2 | 5.9 | 4.2 | 10.5 | 5.0 | 6.30 |
| **Iron*** | *0.78* | *0.53* | 0.66 | 0.50 | 0.86 | 0.69 | 2.43 | 1.00 | 1.64 | 0.47 | 0.52 | 0.70 | 0.51 | 0.47 | 0.62 | 1.00 | 0.66 | 1.06 | 0.13 | 0.27 |
| **US (%)** | *0.91* | *0.10* | 97 | 100 | 93 | 93 | 86 | 92 | 91 | 99 | 69 | 98 | 100 | 80 | 68 | 100 | 97 | 100 | 98 | 77 |
| **SS (%)** | *9* | *0.10* | 3 | 0 | 7 | 7 | 14 | 8 | 9 | 1 | 32 | 2 | 0 | 20 | 32 | 0 | 3 | 0 | 2 | 23 |
| **Width (m)** | *3.7* | *1.96* | 4.08 | 9.94 | 4.70 | 1.86 | 1.51 | 4.40 | 1.54 | 2.63 | 3.03 | 2.21 | 3.81 | 4.02 | 2.98 | 2.88 | 3.21 | 5.31 | 3.17 | 5.69 |
| **Depth(m)** | *0.33* | *0.079* | 40.29 | 49.51 | 20.29 | 15.73 | 17.30 | 23.93 | 13.42 | 26.56 | 28.67 | 40.24 | 32.16 | 31.49 | 31.51 | 27.27 | 27.20 | 46.18 | 22.11 | 45.00 |
| **Flow (rps)** | *216.53* | *126.12* | 184.11 | 307.22 | 307.89 | 20.00 | 258.89 | 333.67 | 162.92 | 248.67 | 338.11 | 322.33 | 340.78 | 252.00 | 408.89 | 64.22 | 217.67 | 27.44 | 79.67 | 23.11 |
| **Riparian FW(m)** | *33.22* | *19.56* | 30 | 0 | 15 | 30 | 30 | 20 | 10 | 30 | 30 | 10 | 15 | 10 | 15 | 15 | 10 | 10 | 12 | 10 |

S1 Table. Mean and standard deviation for the environmental variables measured in the 18 streams of sub-basins Piracanjuba, Ribeirão Vermelho, and Rio dos Bois in the municipality of Silvânia, State of Goiás, Brazil. DO = Dissolved Oxygen, Chl-a = Chlorophyll-a, BOD = biochemical oxygen demand, ORP = oxidation-reduction potential, TDS = total dissolved solids, T. Solids = Total Solids, T. Nitrogen = Total Nitrogen, A. Nitrogen = Ammoniacal Nitrogen, T. Phosphorus = Total Phosphorus, T. Org. Carbon = Total Organic Carbon, US = Unstable Substrate, SS = Stable Substrate, Width (m), and Riparian FW = Riparian Forest Width.

* = mg L-1; # = µg L-1

S2 Table. Abundance (fish) and density (phytoplankton, periphyton and zooplankton) of species measured in the 18 streams of Piracanjuba, Ribeiräo Vermelho, and River dos Bois, in the municipality of Silvânia, State of Goiás, Brazil.

| Species | Total Abundance | P1 | P2 | P3 | P4 | P5 | P6 | P7 | P8 | P9 | P10 | P11 | P12 | P13 | P14 | P15 | P16 | P17 | P18 |
| --- | --- | --- | --- | --- | --- | --- | --- | --- | --- | --- | --- | --- | --- | --- | --- | --- | --- | --- | --- |
| Fish |  |  |  |  |  |  |  |  |  |  |  |  |  |  |  |  |  |  |  |
| *Aspidoras fuscoguttatus* | **1** | **0** | **0** | **0** | **0** | **0** | **1** | **0** | **0** | **0** | **0** | **0** | **0** | **0** | **0** | **0** | **0** | **0** | **0** |
| *Astyanax altiparanae* | 76 | 0 | 0 | 0 | 0 | 17 | 2 | 0 | 0 | 2 | 7 | 18 | 20 | 0 | 4 | 0 | 4 | 2 | 0 |
| *Astyanax bockmanni* | 3 | 0 | 0 | 0 | 0 | 0 | 0 | 0 | 2 | 0 | 0 | 0 | 0 | 0 | 0 | 0 | 0 | 1 | 0 |
| *Astyanax fasciatus* | 57 | 0 | 0 | 0 | 25 | 1 | 0 | 0 | 1 | 0 | 6 | 5 | 12 | 0 | 2 | 4 | 1 | 0 | 0 |
| *Astyanax fasciatus* | 21 | 0 | 0 | 21 | 0 | 0 | 0 | 0 | 0 | 0 | 0 | 0 | 0 | 0 | 0 | 0 | 0 | 0 | 0 |
| *Bryconamericus stramineus* | 31 | 0 | 0 | 0 | 0 | 0 | 0 | 0 | 0 | 1 | 2 | 0 | 0 | 6 | 0 | 21 | 1 | 0 | 0 |
| *Bryconamericus turiuba* | 199 | 2 | 10 | 83 | 0 | 26 | 13 | 0 | 1 | 3 | 5 | 2 | 34 | 2 | 0 | 6 | 3 | 9 | 0 |
| *Cetopsis gobioides* | 2 | 0 | 0 | 0 | 0 | 0 | 2 | 0 | 0 | 0 | 0 | 0 | 0 | 0 | 0 | 0 | 0 | 0 | 0 |
| *Cetopsorhamdia iheringi* | 14 | 0 | 4 | 0 | 0 | 0 | 6 | 0 | 0 | 0 | 1 | 0 | 0 | 2 | 0 | 1 | 0 | 0 | 0 |
| *Characidium gomesi* | 66 | 0 | 0 | 0 | 0 | 0 | 7 | 0 | 2 | 6 | 4 | 17 | 6 | 13 | 0 | 9 | 1 | 0 | 1 |
| *Characidium zebra* | 27 | 0 | 0 | 0 | 0 | 0 | 0 | 0 | 0 | 0 | 2 | 9 | 14 | 0 | 0 | 0 | 2 | 0 | 0 |
| *Cichlasoma paranaense* | 3 | 0 | 2 | 0 | 0 | 0 | 0 | 0 | 0 | 0 | 0 | 0 | 0 | 0 | 0 | 1 | 0 | 0 | 0 |
| *Eigenmannia virescens* | 1 | 0 | 0 | 0 | 0 | 0 | 0 | 0 | 0 | 0 | 0 | 0 | 0 | 1 | 0 | 0 | 0 | 0 | 0 |
| *Gymnotus aff carapo* | 39 | 3 | 1 | 0 | 6 | 4 | 1 | 1 | 2 | 5 | 3 | 4 | 3 | 0 | 2 | 0 | 1 | 2 | 1 |
| *Hasemania hanseni* | 11 | 0 | 0 | 0 | 0 | 0 | 0 | 0 | 0 | 3 | 0 | 8 | 0 | 0 | 0 | 0 | 0 | 0 | 0 |
| *Hisonotus insperatus* | 3 | 0 | 0 | 0 | 0 | 0 | 0 | 0 | 0 | 0 | 3 | 0 | 0 | 0 | 0 | 0 | 0 | 0 | 0 |
| *Hisonotus sp.* | 4 | 0 | 3 | 0 | 0 | 0 | 0 | 0 | 0 | 0 | 0 | 0 | 0 | 0 | 0 | 0 | 0 | 1 | 0 |
| *Hoplias malabaricus* | 2 | 0 | 0 | 0 | 1 | 0 | 0 | 0 | 0 | 0 | 1 | 0 | 0 | 0 | 0 | 0 | 0 | 0 | 0 |
| *Hypostomus ancistroides* | 1 | 0 | 0 | 0 | 0 | 0 | 1 | 0 | 0 | 0 | 0 | 0 | 0 | 0 | 0 | 0 | 0 | 0 | 0 |
| *Hypostomus sp..* | 24 | 1 | 2 | 0 | 0 | 0 | 2 | 0 | 0 | 0 | 2 | 8 | 3 | 3 | 0 | 0 | 3 | 0 | 0 |
| *Imparfinis schubarti* | 99 | 0 | 0 | 0 | 0 | 0 | 1 | 0 | 0 | 0 | 1 | 66 | 6 | 17 | 0 | 7 | 1 | 0 | 0 |
| *Leporinus sp.* | 4 | 1 | 1 | 0 | 0 | 0 | 0 | 0 | 0 | 0 | 0 | 1 | 1 | 0 | 0 | 0 | 0 | 0 | 0 |
| *Leporinus sp.1* | 3 | 0 | 0 | 0 | 0 | 0 | 0 | 0 | 0 | 0 | 0 | 3 | 0 | 0 | 0 | 0 | 0 | 0 | 0 |
| *Parodon nasus* | 3 | 0 | 0 | 0 | 0 | 0 | 0 | 0 | 0 | 0 | 0 | 2 | 1 | 0 | 0 | 0 | 0 | 0 | 0 |
| *Phenacorhamdia tenebrosa* | 24 | 2 | 2 | 0 | 0 | 0 | 2 | 0 | 0 | 1 | 2 | 5 | 0 | 1 | 0 | 9 | 0 | 0 | 0 |
| *Piabina argentea* | 132 | 0 | 2 | 9 | 2 | 0 | 16 | 0 | 10 | 0 | 7 | 0 | 1 | 3 | 9 | 52 | 0 | 21 | 0 |
| *Pimelodela gracilis* | 104 | 0 | 0 | 0 | 0 | 0 | 0 | 0 | 0 | 0 | 0 | 71 | 23 | 1 | 1 | 5 | 3 | 0 | 0 |
| *Pimelodus sp.* | 2 | 0 | 0 | 0 | 0 | 0 | 0 | 0 | 0 | 0 | 0 | 0 | 0 | 0 | 0 | 0 | 2 | 0 | 0 |
| *Poecilia reticulata* | 110 | 0 | 0 | 0 | 5 | 21 | 0 | 62 | 0 | 0 | 0 | 0 | 0 | 0 | 0 | 0 | 0 | 22 | 0 |
| *Prochilodus lineatus* | 2 | 0 | 0 | 0 | 0 | 0 | 0 | 0 | 0 | 0 | 0 | 0 | 0 | 0 | 0 | 0 | 2 | 0 | 0 |
| *Pseudopimelodus pulcher* | 13 | 0 | 0 | 0 | 0 | 0 | 0 | 0 | 0 | 0 | 0 | 0 | 0 | 3 | 0 | 3 | 7 | 0 | 0 |
| *Rhamdia quelen* | 15 | 0 | 0 | 1 | 1 | 2 | 0 | 0 | 3 | 5 | 0 | 1 | 1 | 1 | 0 | 0 | 0 | 0 | 0 |
| *Steindachnerina insculpta* | 1 | 0 | 0 | 0 | 0 | 1 | 0 | 0 | 0 | 0 | 0 | 0 | 0 | 0 | 0 | 0 | 0 | 0 | 0 |
| *Synbranchus marmoratus* | 1 | 0 | 1 | 0 | 0 | 0 | 0 | 0 | 0 | 0 | 0 | 0 | 0 | 0 | 0 | 0 | 0 | 0 | 0 |
| Zooplankton | **Total Density** | **P1** | **P2** | **P3** | **P4** | **P5** | **P6** | **P7** | **P8** | **P9** | **P10** | **P11** | **P12** | **P13** | **P14** | **P15** | **P16** | **P17** | **P18** |
| *Alona affinis* | 5 | 0 | 0 | 2 | 3 | 0 | 0 | 0 | 0 | 0 | 0 | 0 | 0 | 0 | 0 | 0 | 0 | 0 | 0 |
| *Alona guttata* | 2 | 0 | 2 | 0 | 0 | 0 | 0 | 0 | 0 | 0 | 0 | 0 | 0 | 0 | 0 | 0 | 0 | 0 | 0 |
| *Alona ossiani* | 26 | 4 | 0 | 19 | 2 | 0 | 0 | 0 | 0 | 0 | 0 | 0 | 0 | 1 | 0 | 0 | 0 | 0 | 0 |
| *Alona verrucosa* | 3 | 0 | 0 | 0 | 2 | 1 | 0 | 0 | 0 | 0 | 0 | 0 | 0 | 0 | 0 | 0 | 0 | 0 | 0 |
| *Alona yara* | 11 | 3 | 3 | 2 | 0 | 0 | 0 | 0 | 0 | 0 | 0 | 0 | 3 | 0 | 0 | 0 | 0 | 0 | 0 |
| *Alonella dadayi* | 3 | 0 | 0 | 0 | 0 | 0 | 0 | 0 | 0 | 0 | 0 | 0 | 3 | 0 | 0 | 0 | 0 | 0 | 0 |
| *Arcella conica* | 21 | 4 | 0 | 1 | 5 | 0 | 2 | 1 | 3 | 2 | 1 | 2 | 0 | 0 | 0 | 0 | 0 | 0 | 0 |
| *Arcella costata* | 5 | 1 | 0 | 0 | 0 | 0 | 0 | 0 | 0 | 2 | 2 | 0 | 0 | 0 | 0 | 0 | 0 | 0 | 0 |
| *Arcella crenulata* | 5 | 0 | 0 | 0 | 0 | 0 | 0 | 1 | 0 | 0 | 2 | 0 | 0 | 0 | 0 | 0 | 0 | 2 | 0 |
| *Arcella dentata* | 8 | 2 | 0 | 0 | 2 | 0 | 0 | 2 | 2 | 0 | 0 | 0 | 0 | 0 | 0 | 0 | 0 | 0 | 0 |
| *Arcella discoides* | 39 | 7 | 4 | 0 | 0 | 2 | 0 | 0 | 2 | 4 | 3 | 0 | 2 | 0 | 2 | 6 | 4 | 0 | 3 |
| *Arcella gibbosa* | 19 | 4 | 0 | 1 | 4 | 0 | 0 | 0 | 2 | 4 | 2 | 0 | 0 | 0 | 2 | 0 | 0 | 0 | 0 |
| *Arcella gibbosa mitriformis* | 5 | 0 | 0 | 0 | 0 | 0 | 1 | 1 | 0 | 1 | 0 | 2 | 0 | 0 | 0 | 0 | 0 | 0 | 0 |
| *Arcella hemisphaerica* | 8 | 4 | 0 | 0 | 0 | 0 | 0 | 1 | 1 | 2 | 0 | 0 | 0 | 0 | 0 | 0 | 0 | 0 | 0 |
| *Arcella hemisphaerica undulata* | 7 | 0 | 0 | 0 | 1 | 0 | 0 | 0 | 0 | 0 | 0 | 0 | 3 | 2 | 0 | 0 | 0 | 1 | 0 |
| *Arcella megastoma* | 2 | 1 | 0 | 0 | 0 | 0 | 0 | 1 | 0 | 0 | 0 | 0 | 0 | 0 | 0 | 0 | 0 | 0 | 0 |
| *Arcella mitrata* | 8 | 1 | 0 | 0 | 0 | 0 | 0 | 2 | 0 | 2 | 0 | 0 | 0 | 2 | 1 | 0 | 0 | 0 | 0 |
| *Arcella vulgaris* | 50 | 5 | 3 | 4 | 4 | 3 | 0 | 4 | 6 | 2 | 3 | 4 | 0 | 0 | 0 | 5 | 4 | 0 | 3 |
| *Arcella vulgaris undulata* | 7 | 0 | 0 | 0 | 2 | 0 | 0 | 0 | 0 | 1 | 0 | 0 | 0 | 1 | 0 | 0 | 0 | 3 | 0 |
| *Bosmina hagmanni* | **53** | **2** | **1** | **3** | **9** | **8** | **2** | **2** | **1** | **3** | **0** | **3** | **0** | **0** | **0** | **0** | **9** | **10** | **0** |
| *Bosminopsis deitersi* | 67 | 0 | 0 | 0 | 12 | 14 | 6 | 4 | 2 | 3 | 0 | 4 | 0 | 0 | 0 | 0 | 10 | 12 | 0 |
| *Brachionus falcatus* | 19 | 2 | 2 | 2 | 8 | 0 | 0 | 5 | 0 | 0 | 0 | 0 | 0 | 0 | 0 | 0 | 0 | 0 | 0 |
| *Brachionus sp.* | 18 | 2 | 3 | 6 | 7 | 0 | 0 | 0 | 0 | 0 | 0 | 0 | 0 | 0 | 0 | 0 | 0 | 0 | 0 |
| *Centropyxis aculeata* | 24 | 0 | 3 | 2 | 6 | 0 | 1 | 3 | 2 | 0 | 2 | 2 | 2 | 0 | 0 | 0 | 1 | 0 | 0 |
| *Centropyxis aerophila* | 5 | 2 | 0 | 0 | 1 | 0 | 0 | 0 | 0 | 0 | 0 | 0 | 0 | 0 | 0 | 0 | 0 | 2 | 0 |
| *Centropyxis cassis* | 9 | 0 | 0 | 0 | 1 | 0 | 1 | 2 | 1 | 3 | 1 | 0 | 0 | 0 | 0 | 0 | 0 | 0 | 0 |
| *Centropyxis constricta* | 19 | 1 | 0 | 0 | 5 | 0 | 0 | 0 | 3 | 0 | 1 | 3 | 1 | 5 | 0 | 0 | 0 | 0 | 0 |
| *Centropyxis discoides* | 21 | 4 | 2 | 2 | 0 | 0 | 0 | 0 | 3 | 3 | 3 | 3 | 0 | 0 | 0 | 0 | 0 | 1 | 0 |
| *Centropyxis ecornis* | 27 | 3 | 0 | 3 | 3 | 0 | 1 | 3 | 4 | 0 | 2 | 2 | 6 | 0 | 0 | 0 | 0 | 0 | 0 |
| *Centropyxis gibba* | 34 | 6 | 0 | 3 | 2 | 0 | 0 | 0 | 4 | 2 | 2 | 2 | 0 | 5 | 1 | 0 | 2 | 5 | 0 |
| *Centropyxis grandis* | 5 | 1 | 0 | 0 | 0 | 1 | 0 | 0 | 0 | 2 | 0 | 0 | 0 | 0 | 1 | 0 | 0 | 0 | 0 |
| *Centropyxis minuta* | 4 | 0 | 0 | 0 | 1 | 2 | 0 | 0 | 1 | 0 | 0 | 0 | 0 | 0 | 0 | 0 | 0 | 0 | 0 |
| *Centropyxis platystoma* | 8 | 2 | 0 | 0 | 0 | 0 | 0 | 2 | 1 | 1 | 0 | 0 | 1 | 0 | 0 | 0 | 0 | 0 | 1 |
| *Cephalodella sp.* | 9 | 0 | 0 | 2 | 0 | 0 | 2 | 2 | 0 | 0 | 0 | 0 | 0 | 0 | 3 | 0 | 0 | 0 | 0 |
| *Ceriodaphnia cornuta* | 20 | 0 | 0 | 2 | 0 | 1 | 3 | 0 | 0 | 4 | 0 | 0 | 5 | 0 | 3 | 0 | 0 | 2 | 0 |
| *Chydorus eurynotus* | 9 | 2 | 4 | 3 | 0 | 0 | 0 | 0 | 0 | 0 | 0 | 0 | 0 | 0 | 0 | 0 | 0 | 0 | 0 |
| *Chydorus pubescens* | 7 | 0 | 4 | 0 | 0 | 1 | 0 | 0 | 0 | 0 | 0 | 0 | 0 | 2 | 0 | 0 | 0 | 0 | 0 |
| *Coronatella poppei* | 9 | 4 | 0 | 2 | 3 | 0 | 0 | 0 | 0 | 0 | 0 | 0 | 0 | 0 | 0 | 0 | 0 | 0 | 0 |
| *Cyclopyxis eurystoma* | 7 | 0 | 0 | 0 | 1 | 0 | 0 | 2 | 1 | 0 | 3 | 0 | 0 | 0 | 0 | 0 | 0 | 0 | 0 |
| *Cyclopyxis kahli* | 12 | 0 | 0 | 0 | 1 | 0 | 0 | 0 | 0 | 0 | 0 | 0 | 4 | 6 | 0 | 0 | 0 | 1 | 0 |
| *Diaphanosoma spinulosum* | 3 | 0 | 1 | 2 | 0 | 0 | 0 | 0 | 0 | 0 | 0 | 0 | 0 | 0 | 0 | 0 | 0 | 0 | 0 |
| *Difflugia achlora* | 11 | 1 | 0 | 0 | 0 | 0 | 0 | 1 | 0 | 2 | 0 | 0 | 0 | 7 | 0 | 0 | 0 | 0 | 0 |
| *Difflugia corona* | 8 | 0 | 0 | 0 | 0 | 0 | 0 | 0 | 0 | 2 | 0 | 0 | 3 | 0 | 0 | 1 | 0 | 2 | 0 |
| *Difflugia cylindrus* | 8 | 0 | 0 | 0 | 0 | 1 | 0 | 1 | 1 | 2 | 0 | 0 | 0 | 0 | 0 | 0 | 0 | 3 | 0 |
| *Difflugia elegans* | 14 | 6 | 0 | 0 | 3 | 0 | 0 | 0 | 0 | 0 | 3 | 0 | 1 | 0 | 1 | 0 | 0 | 0 | 0 |
| *Difflugia gramen* | 0 | 0 | 0 | 0 | 0 | 0 | 0 | 0 | 0 | 0 | 0 | 0 | 0 | 0 | 0 | 0 | 0 | 0 | 0 |
| *Difflugia lobostoma* | 13 | 4 | 0 | 0 | 2 | 0 | 0 | 0 | 2 | 1 | 0 | 2 | 0 | 2 | 0 | 0 | 0 | 0 | 0 |
| *Difflugia oblonga* | 21 | 0 | 2 | 0 | 0 | 0 | 1 | 2 | 0 | 0 | 0 | 4 | 5 | 0 | 0 | 0 | 2 | 5 | 0 |
| *Euglypha acanthophora* | 22 | 0 | 0 | 0 | 0 | 1 | 0 | 1 | 3 | 0 | 1 | 4 | 0 | 4 | 3 | 0 | 0 | 4 | 1 |
| *Euglypha denticulata* | 7 | 2 | 0 | 0 | 0 | 0 | 0 | 0 | 0 | 1 | 0 | 4 | 0 | 0 | 0 | 0 | 0 | 0 | 0 |
| *Euglypha filifera* | 4 | 0 | 0 | 0 | 1 | 0 | 0 | 1 | 0 | 0 | 0 | 0 | 2 | 0 | 0 | 0 | 0 | 0 | 0 |
| *Euglypha laevis* | 12 | 2 | 0 | 0 | 0 | 0 | 0 | 3 | 1 | 3 | 2 | 0 | 0 | 0 | 0 | 0 | 0 | 1 | 0 |
| *Euglypha rotunda* | 11 | 0 | 0 | 0 | 2 | 0 | 0 | 0 | 0 | 0 | 0 | 2 | 1 | 3 | 0 | 2 | 0 | 1 | 0 |
| *Euglypha sp.* | 6 | 2 | 0 | 0 | 0 | 2 | 0 | 0 | 1 | 0 | 0 | 0 | 0 | 0 | 0 | 0 | 0 | 1 | 0 |
| *Euglypha tuberculata* | 16 | 0 | 0 | 0 | 3 | 0 | 0 | 2 | 0 | 0 | 1 | 0 | 4 | 3 | 3 | 0 | 0 | 0 | 0 |
| *Euryalona brasiliensis* | 5 | 0 | 3 | 0 | 2 | 0 | 0 | 0 | 0 | 0 | 0 | 0 | 0 | 0 | 0 | 0 | 0 | 0 | 0 |
| *Heleopera petricola* | 21 | 5 | 0 | 2 | 2 | 4 | 0 | 0 | 1 | 2 | 0 | 0 | 0 | 1 | 0 | 1 | 0 | 3 | 0 |
| *Keratella americana* | 24 | 4 | 3 | 6 | 0 | 0 | 0 | 5 | 0 | 0 | 0 | 0 | 4 | 2 | 0 | 0 | 0 | 0 | 0 |
| *Keratella cochlearis* | 11 | 0 | 0 | 5 | 6 | 0 | 0 | 0 | 0 | 0 | 0 | 0 | 0 | 0 | 0 | 0 | 0 | 0 | 0 |
| *Keratella hispida* | 14 | 3 | 0 | 0 | 5 | 0 | 0 | 6 | 0 | 0 | 0 | 0 | 0 | 0 | 0 | 0 | 0 | 0 | 0 |
| *Keratella lenzi* | 15 | 0 | 2 | 5 | 0 | 0 | 0 | 6 | 0 | 0 | 0 | 0 | 0 | 2 | 0 | 0 | 0 | 0 | 0 |
| *Lecane bulla* | 22 | 0 | 2 | 0 | 4 | 0 | 4 | 0 | 0 | 0 | 0 | 2 | 0 | 1 | 4 | 0 | 0 | 0 | 5 |
| *Lecane curvicornis* | 8 | 4 | 0 | 2 | 0 | 0 | 2 | 0 | 0 | 0 | 0 | 0 | 0 | 0 | 0 | 0 | 0 | 0 | 0 |
| *Lecane papuana* | 14 | 4 | 0 | 0 | 2 | 0 | 0 | 1 | 0 | 0 | 0 | 0 | 2 | 0 | 0 | 0 | 0 | 2 | 3 |
| *Lecane quadridentata* | 7 | 0 | 2 | 2 | 0 | 0 | 0 | 0 | 0 | 0 | 0 | 0 | 0 | 0 | 3 | 0 | 0 | 0 | 0 |
| *Lecane signifera* | 13 | 0 | 2 | 0 | 0 | 0 | 4 | 0 | 0 | 0 | 0 | 3 | 0 | 0 | 0 | 0 | 0 | 0 | 4 |
| *Lepadella sp.* | 20 | 3 | 0 | 0 | 3 | 0 | 2 | 0 | 0 | 0 | 0 | 3 | 3 | 2 | 0 | 0 | 0 | 0 | 4 |
| *Lesquereusia modesta* | 15 | 2 | 0 | 0 | 0 | 3 | 0 | 0 | 0 | 0 | 2 | 1 | 3 | 0 | 2 | 0 | 0 | 2 | 0 |
| *Lesquereusia spiralis* | 11 | 4 | 0 | 0 | 2 | 0 | 2 | 0 | 0 | 3 | 0 | 0 | 0 | 0 | 0 | 0 | 0 | 0 | 0 |
| *Limnocalanus sp.* | 28 | 0 | 2 | 2 | 6 | 0 | 0 | 0 | 2 | 1 | 0 | 1 | 0 | 0 | 2 | 0 | 4 | 6 | 2 |
| *Macrothrix sp.* | 7 | 3 | 2 | 0 | 0 | 0 | 0 | 0 | 0 | 0 | 0 | 0 | 0 | 2 | 0 | 0 | 0 | 0 | 0 |
| *Moina micrura* | 77 | 2 | 0 | 73 | 2 | 0 | 0 | 0 | 0 | 0 | 0 | 0 | 0 | 0 | 0 | 0 | 0 | 0 | 0 |
| *Moina minuta* | 6 | 3 | 0 | 3 | 0 | 0 | 0 | 0 | 0 | 0 | 0 | 0 | 0 | 0 | 0 | 0 | 0 | 0 | 0 |
| *Nebela sp.* | 4 | 0 | 0 | 0 | 0 | 0 | 0 | 4 | 0 | 0 | 0 | 0 | 0 | 0 | 0 | 0 | 0 | 0 | 0 |
| *Netzelia oviformis* | 9 | 0 | 0 | 0 | 0 | 0 | 0 | 3 | 2 | 0 | 0 | 0 | 1 | 0 | 0 | 0 | 0 | 3 | 0 |
| *Netzelia tuberculata* | 12 | 7 | 0 | 0 | 0 | 0 | 0 | 0 | 0 | 0 | 0 | 1 | 0 | 3 | 1 | 0 | 0 | 0 | 0 |
| *Netzelia wailesi* | 9 | 0 | 0 | 0 | 2 | 0 | 0 | 0 | 0 | 0 | 1 | 0 | 6 | 0 | 0 | 0 | 0 | 0 | 0 |
| *Nicsmirnovius paggii* | 5 | 0 | 0 | 3 | 0 | 0 | 0 | 0 | 0 | 0 | 0 | 0 | 2 | 0 | 0 | 0 | 0 | 0 | 0 |
| *Notodiaptomus sp. 1* | 23 | 0 | 0 | 0 | 3 | 3 | 1 | 2 | 1 | 0 | 0 | 0 | 2 | 0 | 4 | 0 | 3 | 4 | 0 |
| *Notodiaptomus sp. 2* | 20 | 0 | 2 | 2 | 0 | 2 | 0 | 1 | 0 | 1 | 0 | 1 | 0 | 0 | 0 | 0 | 7 | 4 | 0 |
| *Protocucurbitella coroniformes* | 6 | 3 | 0 | 0 | 1 | 0 | 0 | 0 | 0 | 1 | 0 | 0 | 1 | 0 | 0 | 0 | 0 | 0 | 0 |
| *Quadrulella symmetrica* | 14 | 0 | 0 | 0 | 4 | 0 | 0 | 0 | 3 | 0 | 0 | 0 | 0 | 2 | 0 | 0 | 3 | 2 | 0 |
| *Thermocyclops minutus* | 52 | 2 | 0 | 3 | 7 | 7 | 2 | 0 | 0 | 4 | 0 | 0 | 3 | 0 | 5 | 0 | 8 | 9 | 2 |
| *Thermocyclops sp.* | 35 | 1 | 0 | 0 | 3 | 6 | 1 | 3 | 4 | 0 | 0 | 0 | 2 | 1 | 2 | 0 | 5 | 7 | 0 |
| *Trichocerca bicristata* | 11 | 4 | 3 | 0 | 0 | 0 | 0 | 0 | 0 | 0 | 0 | 0 | 0 | 0 | 0 | 0 | 0 | 1 | 3 |
| *Trichocerca similis* | 7 | 0 | 3 | 0 | 2 | 0 | 2 | 0 | 0 | 0 | 0 | 0 | 0 | 0 | 0 | 0 | 0 | 0 | 0 |
| *Trichocerca sp.* | 16 | 2 | 0 | 2 | 0 | 0 | 0 | 0 | 0 | 0 | 0 | 2 | 6 | 1 | 2 | 0 | 0 | 0 | 1 |
| *Trinema enchelys* | 18 | 7 | 0 | 0 | 0 | 0 | 0 | 2 | 1 | 2 | 0 | 2 | 0 | 0 | 3 | 0 | 0 | 0 | 1 |
| Periphyton | **Total Density** | **P1** | **P2** | **P3** | **P4** | **P5** | **P6** | **P7** | **P8** | **P9** | **P10** | **P11** | **P12** | **P13** | **P14** | **P15** | **P16** | **P17** | **P18** |
| *Amphipleura sp.1* | **16** | **0** | **0** | **0** | **0** | **0** | **0** | **0** | **0** | **0** | **0** | **16** | **0** | **0** | **0** | **0** | **0** | **0** | **0** |
| *Aulacoseira sp.1* | 1 | 0 | 0 | 0 | 1 | 0 | 0 | 0 | 0 | 0 | 0 | 0 | 0 | 0 | 0 | 0 | 0 | 0 | 0 |
| *Aulacoseira sp.2* | 2 | 0 | 0 | 0 | 0 | 0 | 0 | 0 | 0 | 0 | 0 | 0 | 0 | 0 | 0 | 0 | 0 | 1 | 1 |
| *Chridella crystiformis* | 30 | 0 | 0 | 20 | 0 | 1 | 9 | 0 | 0 | 0 | 0 | 0 | 0 | 0 | 0 | 0 | 0 | 0 | 0 |
| *Closterium leibleinii* | 2 | 0 | 0 | 0 | 1 | 1 | 0 | 0 | 0 | 0 | 0 | 0 | 0 | 0 | 0 | 0 | 0 | 0 | 0 |
| *Coelastrum reticulatum* | 1 | 0 | 0 | 0 | 0 | 0 | 0 | 0 | 0 | 1 | 0 | 0 | 0 | 0 | 0 | 0 | 0 | 0 | 0 |
| *Cosmarium sp.1* | 1 | 0 | 1 | 0 | 0 | 0 | 0 | 0 | 0 | 0 | 0 | 0 | 0 | 0 | 0 | 0 | 0 | 0 | 0 |
| *Cosmarium sp.2* | 0 | 0 | 0 | 0 | 0 | 0 | 0 | 0 | 0 | 0 | 0 | 0 | 0 | 0 | 0 | 0 | 0 | 0 | 0 |
| *Cosmarium sp.3* | 2 | 0 | 0 | 0 | 0 | 0 | 0 | 1 | 0 | 0 | 0 | 0 | 0 | 0 | 0 | 1 | 0 | 0 | 0 |
| *Cyclotella sp.1* | 101 | 0 | 0 | 100 | 0 | 0 | 0 | 0 | 0 | 0 | 0 | 0 | 0 | 0 | 0 | 0 | 0 | 1 | 0 |
| Taxon sp.1 | 106 | 0 | 0 | 13 | 0 | 0 | 30 | 0 | 0 | 0 | 62 | 0 | 0 | 0 | 1 | 0 | 0 | 0 | 0 |
| *Eremosphaera sp.1* | 1 | 0 | 0 | 0 | 1 | 0 | 0 | 0 | 0 | 0 | 0 | 0 | 0 | 0 | 0 | 0 | 0 | 0 | 0 |
| *Eunotia sp.1* | 76 | 0 | 4 | 0 | 0 | 0 | 0 | 1 | 0 | 14 | 0 | 38 | 7 | 0 | 0 | 0 | 0 | 10 | 2 |
| *Eunotia sp.3* | 96 | 0 | 0 | 0 | 0 | 1 | 1 | 20 | 0 | 0 | 0 | 36 | 5 | 4 | 1 | 4 | 1 | 23 | 0 |
| *Eunotia sp.4* | 4 | 0 | 0 | 0 | 0 | 0 | 0 | 0 | 0 | 0 | 1 | 1 | 0 | 0 | 0 | 0 | 2 | 0 | 0 |
| *Eunotia sp.6* | 1 | 0 | 0 | 0 | 0 | 0 | 0 | 0 | 0 | 0 | 0 | 0 | 0 | 0 | 0 | 1 | 0 | 0 | 0 |
| *Fragilaria sp.1* | 0 | 0 | 0 | 0 | 0 | 0 | 0 | 0 | 0 | 0 | 0 | 0 | 0 | 0 | 0 | 0 | 0 | 0 | 0 |
| *Frustulia sp.1* | 1 | 0 | 0 | 0 | 0 | 0 | 0 | 1 | 0 | 0 | 0 | 0 | 0 | 0 | 0 | 0 | 0 | 0 | 0 |
| *Geitlerinema sp.2* | 23 | 0 | 8 | 15 | 0 | 0 | 0 | 0 | 0 | 0 | 0 | 0 | 0 | 0 | 0 | 0 | 0 | 0 | 0 |
| *Gloeocapsopsis sp.1* | 116 | 0 | 0 | 16 | 100 | 0 | 0 | 0 | 0 | 0 | 0 | 0 | 0 | 0 | 0 | 0 | 0 | 0 | 0 |
| *Gokenkinia radiata* | 0 | 0 | 0 | 0 | 0 | 0 | 0 | 0 | 0 | 0 | 0 | 0 | 0 | 0 | 0 | 0 | 0 | 0 | 0 |
| *Gyrosigma sp.1* | 2 | 0 | 0 | 0 | 0 | 0 | 0 | 0 | 0 | 0 | 0 | 2 | 0 | 0 | 0 | 0 | 0 | 0 | 0 |
| *Homoeothrix sp.1* | 100 | 0 | 100 | 0 | 0 | 0 | 0 | 0 | 0 | 0 | 0 | 0 | 0 | 0 | 0 | 0 | 0 | 0 | 0 |
| *Monoraphidium contortum* | 1 | 0 | 0 | 0 | 0 | 0 | 0 | 0 | 0 | 0 | 1 | 0 | 0 | 0 | 0 | 0 | 0 | 0 | 0 |
| *Monoraphidiumarcuatum* | 2 | 0 | 0 | 0 | 0 | 0 | 0 | 0 | 0 | 0 | 0 | 0 | 0 | 0 | 0 | 1 | 0 | 1 | 0 |
| *Monoraphidiumgriffithii* | 3 | 0 | 0 | 0 | 0 | 0 | 0 | 0 | 0 | 0 | 0 | 0 | 3 | 0 | 0 | 0 | 0 | 0 | 0 |
| *Navicula 1* | 165 | 0 | 2 | 0 | 1 | 10 | 2 | 0 | 0 | 1 | 6 | 100 | 9 | 21 | 3 | 4 | 2 | 4 | 0 |
| *Nitzschia sp.1* | 4 | 0 | 0 | 0 | 0 | 0 | 0 | 0 | 0 | 0 | 4 | 0 | 0 | 0 | 0 | 0 | 0 | 0 | 0 |
| *Peridinium sp.2* | 3 | 0 | 0 | 0 | 0 | 0 | 0 | 0 | 0 | 3 | 0 | 0 | 0 | 0 | 0 | 0 | 0 | 0 | 0 |
| *Phormidium sp.1* | 85 | 0 | 85 | 0 | 0 | 0 | 0 | 0 | 0 | 0 | 0 | 0 | 0 | 0 | 0 | 0 | 0 | 0 | 0 |
| *Pinnularia latarea* | 1 | 0 | 0 | 0 | 0 | 0 | 0 | 0 | 0 | 0 | 0 | 0 | 0 | 0 | 0 | 0 | 1 | 0 | 0 |
| *Pinnularia saprophila* | 3 | 0 | 0 | 0 | 0 | 0 | 0 | 0 | 0 | 0 | 0 | 0 | 0 | 0 | 0 | 0 | 0 | 3 | 0 |
| *Pinnularia sp.1* | 1 | 0 | 0 | 1 | 0 | 0 | 0 | 0 | 0 | 0 | 0 | 0 | 0 | 0 | 0 | 0 | 0 | 0 | 0 |
| *Pseudonabaena limnetica* | 140 | 0 | 0 | 60 | 0 | 55 | 13 | 6 | 1 | 0 | 4 | 0 | 0 | 0 | 0 | 1 | 0 | 0 | 0 |
| *Rhizoclonium sp.1* | 3 | 0 | 3 | 0 | 0 | 0 | 0 | 0 | 0 | 0 | 0 | 0 | 0 | 0 | 0 | 0 | 0 | 0 | 0 |
| *Scenedesmus opoliensis* | 1 | 0 | 0 | 1 | 0 | 0 | 0 | 0 | 0 | 0 | 0 | 0 | 0 | 0 | 0 | 0 | 0 | 0 | 0 |
| *Sellaphora sp.1* | 9 | 0 | 0 | 0 | 0 | 1 | 0 | 3 | 0 | 0 | 4 | 0 | 0 | 1 | 0 | 0 | 0 | 0 | 0 |
| *Sellaphora sp.2* | 197 | 0 | 0 | 0 | 0 | 0 | 0 | 100 | 0 | 0 | 0 | 42 | 22 | 2 | 0 | 14 | 0 | 17 | 0 |
| *Sellaphora sp.3* | 8 | 0 | 0 | 0 | 0 | 0 | 0 | 0 | 0 | 0 | 0 | 0 | 0 | 0 | 0 | 1 | 0 | 7 | 0 |
| *Spirogyra sp.1* | 1 | 0 | 0 | 1 | 0 | 0 | 0 | 0 | 0 | 0 | 0 | 0 | 0 | 0 | 0 | 0 | 0 | 0 | 0 |
| *Unaria una* | 4 | 0 | 0 | 0 | 0 | 0 | 0 | 0 | 0 | 0 | 0 | 0 | 0 | 3 | 0 | 1 | 0 | 0 | 0 |
| *Xanthonema sp.1* | 1 | 0 | 1 | 0 | 0 | 0 | 0 | 0 | 0 | 0 | 0 | 0 | 0 | 0 | 0 | 0 | 0 | 0 | 0 |
| Phytoplankton | **Total Density** | **P1** | **P2** | **P3** | **P4** | **P5** | **P6** | **P7** | **P8** | **P9** | **P10** | **P11** | **P12** | **P13** | **P14** | **P15** | **P16** | **P17** | **P18** |
| *Amphipleura sp.1* | 5.59 | 0 | 0 | 0 | 0 | 0 | 0 | 0 | 0 | 0 | 0 | 5.59 | 0 | 0 | 0 | 0 | 0 | 0 | 0 |
| *Aulacoseira sp.1* | **11.18** | **0** | **0** | **0** | **0** | **5.59** | **0** | **0** | **0** | **0** | **0** | **0** | **0** | **0** | **0** | **0** | **0** | **0** | **5.59** |
| *Chlamydomonas sp.1* | 33.55 | 0 | 0 | 0 | 0 | 5.59 | 0 | 0 | 0 | 0 | 0 | 5.59 | 0 | 0 | 0 | 0 | 0 | 0 | 22.36 |
| *Chlamydomonas sp.2* | 11.18 | 0 | 0 | 0 | 0 | 0 | 0 | 0 | 0 | 0 | 0 | 0 | 11.18 | 0 | 0 | 0 | 0 | 0 | 0 |
| *Chrococcus minimus* | 352.24 | 0 | 0 | 0 | 0 | 0 | 0 | 0 | 0 | 0 | 0 | 0 | 0 | 0 | 0 | 0 | 0 | 0 | 352.24 |
| *Chrococcus minutus* | 83.87 | 67.09 | 0 | 5.59 | 0 | 5.59 | 0 | 0 | 0 | 0 | 0 | 5.59 | 0 | 0 | 0 | 0 | 0 | 0 | 0 |
| *Closterium leibleinii* | 5.59 | 0 | 0 | 0 | 0 | 0 | 0 | 0 | 0 | 0 | 0 | 5.59 | 0 | 0 | 0 | 0 | 0 | 0 | 0 |
| *Coelastrum reticulatum* | 5.59 | 0 | 0 | 0 | 0 | 0 | 0 | 0 | 0 | 0 | 0 | 0 | 0 | 0 | 0 | 0 | 0 | 0 | 5.59 |
| *Cosmarium sp.1* | 5.59 | 0 | 0 | 0 | 0 | 0 | 0 | 5.59 | 0 | 0 | 0 | 0 | 0 | 0 | 0 | 0 | 0 | 0 | 0 |
| *Cosmarium sp.2* | 11.18 | 0 | 0 | 0 | 0 | 0 | 0 | 0 | 0 | 0 | 0 | 0 | 0 | 0 | 0 | 11.18 | 0 | 0 | 0 |
| *Crucigenia quatrata* | 5.59 | 0 | 0 | 0 | 0 | 0 | 0 | 0 | 0 | 0 | 0 | 0 | 5.59 | 0 | 0 | 0 | 0 | 0 | 0 |
| *Crucigenia tetrapedia* | 11.18 | 11.18 | 0 | 0 | 0 | 0 | 0 | 0 | 0 | 0 | 0 | 0 | 0 | 0 | 0 | 0 | 0 | 0 | 0 |
| *Cryptomonas erosa* | 33.55 | 0 | 0 | 0 | 0 | 5.59 | 0 | 0 | 0 | 5.59 | 0 | 0 | 0 | 0 | 11.18 | 11.18 | 0 | 0 | 0 |
| *Cryptomonas marsonii* | 67.09 | 0 | 0 | 0 | 0 | 0 | 0 | 0 | 0 | 0 | 5.59 | 0 | 0 | 61.50 | 0 | 0 | 0 | 0 | 0 |
| *Cryptomonas obovata* | 33.55 | 0 | 0 | 0 | 0 | 0 | 0 | 16.77 | 5.59 | 0 | 5.59 | 5.59 | 0 | 0 | 0 | 0 | 0 | 0 | 0 |
| *Cryptomonas pierenoidifera* | 5.59 | 0 | 0 | 0 | 0 | 0 | 5.59 | 0 | 0 | 0 | 0 | 0 | 0 | 0 | 0 | 0 | 0 | 0 | 0 |
| *Dictyosphaerium pulchellum* | 5.59 | 0 | 0 | 5.59 | 0 | 0 | 0 | 0 | 0 | 0 | 0 | 0 | 0 | 0 | 0 | 0 | 0 | 0 | 0 |
| *Dinobryon elegantissimum* | 5.59 | 0 | 0 | 0 | 0 | 0 | 0 | 0 | 0 | 0 | 5.59 | 0 | 0 | 0 | 0 | 0 | 0 | 0 | 0 |
| *Eremosphaera sp.1* | 301.92 | 268.37 | 0 | 5.59 | 0 | 5.59 | 0 | 0 | 0 | 0 | 5.59 | 0 | 0 | 0 | 0 | 5.59 | 0 | 0 | 11.18 |
| *Euglena limnophila* | 5.59 | 0 | 0 | 0 | 0 | 5.59 | 0 | 0 | 0 | 0 | 0 | 0 | 0 | 0 | 0 | 0 | 0 | 0 | 0 |
| *Eunotia sp.1* | 33.55 | 0 | 0 | 0 | 0 | 0 | 0 | 0 | 0 | 0 | 0 | 0 | 0 | 0 | 0 | 16.77 | 0 | 0 | 16.77 |
| *Eunotia sp.2* | 11.18 | 0 | 0 | 0 | 0 | 0 | 0 | 0 | 0 | 0 | 0 | 5.59 | 0 | 0 | 0 | 0 | 0 | 0 | 5.59 |
| *Eunotia sp.3* | 123.00 | 11.18 | 0 | 0 | 5.59 | 5.59 | 0 | 5.59 | 0 | 0 | 5.59 | 0 | 0 | 5.59 | 5.59 | 55.91 | 22.36 | 0 | 0 |
| *Eunotia sp.4* | 33.55 | 0 | 0 | 0 | 0 | 16.77 | 0 | 0 | 0 | 0 | 0 | 5.59 | 5.59 | 0 | 5.59 | 0 | 0 | 0 | 0 |
| *Eunotia sp.5* | 11.18 | 0 | 0 | 0 | 0 | 0 | 0 | 0 | 0 | 0 | 0 | 0 | 0 | 0 | 0 | 11.18 | 0 | 0 | 0 |
| *Franceia droescheri* | 5.59 | 0 | 0 | 0 | 0 | 0 | 0 | 5.59 | 0 | 0 | 0 | 0 | 0 | 0 | 0 | 0 | 0 | 0 | 0 |
| *Frustulia sp.1* | 39.14 | 0 | 0 | 0 | 0 | 5.59 | 0 | 0 | 0 | 0 | 0 | 5.59 | 0 | 0 | 0 | 27.96 | 0 | 0 | 0 |
| *Geitlerinema sp.1* | 16.77 | 0 | 0 | 11.18 | 5.59 | 0 | 0 | 0 | 0 | 0 | 0 | 0 | 0 | 0 | 0 | 0 | 0 | 0 | 0 |
| *Gokenkinia radiata* | 16.77 | 0 | 0 | 11.18 | 0 | 5.59 | 0 | 0 | 0 | 0 | 0 | 0 | 0 | 0 | 0 | 0 | 0 | 0 | 0 |
| *Golenkinia sp.1* | 5.59 | 0 | 0 | 0 | 0 | 5.59 | 0 | 0 | 0 | 0 | 0 | 0 | 0 | 0 | 0 | 0 | 0 | 0 | 0 |
| *Kirchneriella sp.1* | 27.96 | 22.36 | 0 | 0 | 0 | 0 | 0 | 5.59 | 0 | 0 | 0 | 0 | 0 | 0 | 0 | 0 | 0 | 0 | 0 |
| *Merismopedia sp.1* | 44.73 | 0 | 0 | 39.14 | 0 | 0 | 0 | 0 | 0 | 0 | 0 | 5.59 | 0 | 0 | 0 | 0 | 0 | 0 | 0 |
| *Monoraphidium arcuatum* | 251.60 | 44.73 | 0 | 27.96 | 22.36 | 5.59 | 5.59 | 0 | 5.59 | 33.55 | 55.91 | 0 | 0 | 11.18 | 0 | 27.96 | 0 | 0 | 11.18 |
| *Monoraphidium caribeum* | 5.59 | 0 | 0 | 0 | 0 | 5.59 | 0 | 0 | 0 | 0 | 0 | 0 | 0 | 0 | 0 | 0 | 0 | 0 | 0 |
| *Monoraphidium contortum* | 212.46 | 67.09 | 0 | 16.77 | 0 | 0 | 5.59 | 0 | 22.36 | 50.32 | 27.96 | 5.59 | 5.59 | 0 | 0 | 0 | 0 | 5.59 | 5.59 |
| *Monoraphidium griffithii* | 1375.40 | 0 | 0 | 0 | 0 | 0 | 5.59 | 0 | 0 | 0 | 0 | 967.25 | 380.19 | 0 | 0 | 0 | 0 | 0 | 22.36 |
| *Monoraphidium irregulare* | 5.59 | 0 | 0 | 0 | 0 | 0 | 0 | 0 | 0 | 0 | 0 | 0 | 0 | 0 | 0 | 0 | 0 | 0 | 5.59 |
| *Monoraphidium komarkovae* | 111.82 | 0 | 0 | 0 | 0 | 33.55 | 0 | 0 | 0 | 0 | 33.55 | 0 | 22.36 | 0 | 0 | 0 | 0 | 22.36 | 0 |
| *Navicula sp.1* | 357.83 | 5.59 | 0 | 0 | 0 | 123.00 | 0 | 11.18 | 0 | 5.59 | 0 | 167.73 | 16.77 | 0 | 11.18 | 16.77 | 0 | 0 | 0 |
| *Nitzschia sp.1* | 251.60 | 0 | 0 | 0 | 0 | 173.32 | 0 | 0 | 0 | 0 | 33.55 | 22.36 | 0 | 0 | 5.59 | 5.59 | 5.59 | 0 | 5.59 |
| *Parvodinium umbonatum* | 16.77 | 0 | 0 | 0 | 0 | 0 | 5.59 | 11.18 | 0 | 0 | 0 | 0 | 0 | 0 | 0 | 0 | 0 | 0 | 0 |
| *Pediastrum gracillimum* | 22.36 | 22.36 | 0 | 0 | 0 | 0 | 0 | 0 | 0 | 0 | 0 | 0 | 0 | 0 | 0 | 0 | 0 | 0 | 0 |
| *Pedinomonas minutissima* | 5.59 | 0 | 0 | 0 | 0 | 0 | 0 | 0 | 0 | 0 | 0 | 0 | 0 | 0 | 0 | 0 | 0 | 5.59 | 0 |
| *Peridinium sp.1* | 150.96 | 0 | 0 | 5.59 | 0 | 0 | 0 | 22.36 | 0 | 0 | 11.18 | 83.87 | 27.96 | 0 | 0 | 0 | 0 | 0 | 0 |
| *Peridinium sp.2* | 11.18 | 0 | 0 | 0 | 0 | 0 | 0 | 11.18 | 0 | 0 | 0 | 0 | 0 | 0 | 0 | 0 | 0 | 0 | 0 |
| *Phacus sp.1* | 22.36 | 0 | 0 | 0 | 0 | 0 | 0 | 0 | 0 | 0 | 0 | 16.77 | 0 | 0 | 0 | 0 | 5.59 | 0 | 0 |
| *Pinnularia sp.1* | 22.36 | 0 | 0 | 0 | 0 | 0 | 0 | 11.18 | 0 | 0 | 0 | 5.59 | 0 | 0 | 0 | 5.59 | 0 | 0 | 0 |
| *Pleurotaenium minutum* | 11.18 | 0 | 0 | 0 | 0 | 0 | 0 | 0 | 0 | 0 | 0 | 0 | 0 | 0 | 0 | 0 | 0 | 0 | 11.18 |
| *Pleurotaenium tenuissimum* | 5.59 | 0 | 0 | 0 | 0 | 0 | 0 | 0 | 0 | 0 | 5.59 | 0 | 0 | 0 | 0 | 0 | 0 | 0 | 0 |
| *Pseudokephryon ovum* | 167.73 | 0 | 0 | 0 | 0 | 106.23 | 0 | 5.59 | 5.59 | 0 | 33.55 | 0 | 5.59 | 11.18 | 0 | 0 | 0 | 0 | 0 |
| *Pseudonabaena limnetica* | 21.68 | 0 | 4.90 | 5.59 | 0 | 0 | 0 | 5.59 | 0 | 0 | 0 | 0 | 0 | 0 | 0 | 5.59 | 0 | 0 | 0 |
| *Scenedesmus denticulatus* | 234.82 | 5.59 | 0 | 223.64 | 0 | 0 | 0 | 0 | 0 | 0 | 0 | 0 | 5.59 | 0 | 0 | 0 | 0 | 0 | 0 |
| *Scenedesmus opoliensis* | 33.55 | 0 | 0 | 11.18 | 0 | 16.77 | 0 | 0 | 5.59 | 0 | 0 | 0 | 0 | 0 | 0 | 0 | 0 | 0 | 0 |
| *Sellaphora sp.1* | 50.32 | 0 | 0 | 0 | 0 | 0 | 0 | 0 | 0 | 0 | 0 | 22.36 | 11.18 | 0 | 0 | 11.18 | 0 | 0 | 5.59 |
| *Sellaphora sp.2* | 273.96 | 0 | 0 | 0 | 0 | 0 | 0 | 0 | 0 | 5.59 | 0 | 123.00 | 39.14 | 0 | 5.59 | 16.77 | 0 | 0 | 83.87 |
| *Staurastrum sp.1* | 67.09 | 67.09 | 0 | 0 | 0 | 0 | 0 | 0 | 0 | 0 | 0 | 0 | 0 | 0 | 0 | 0 | 0 | 0 | 0 |
| *Staurastrum sp.2* | 5.59 | 5.59 | 0 | 0 | 0 | 0 | 0 | 0 | 0 | 0 | 0 | 0 | 0 | 0 | 0 | 0 | 0 | 0 | 0 |
| *Staurastrum sp.3* | 5.59 | 5.59 | 0 | 0 | 0 | 0 | 0 | 0 | 0 | 0 | 0 | 0 | 0 | 0 | 0 | 0 | 0 | 0 | 0 |
| *Staurastrum sp.4* | 5.59 | 5.59 | 0 | 0 | 0 | 0 | 0 | 0 | 0 | 0 | 0 | 0 | 0 | 0 | 0 | 0 | 0 | 0 | 0 |
| *Staurastrum sp.5* | 5.59 | 0 | 0 | 5.59 | 0 | 0 | 0 | 0 | 0 | 0 | 0 | 0 | 0 | 0 | 0 | 0 | 0 | 0 | 0 |
| *Staurastrum sp.6* | 5.59 | 0 | 0 | 5.59 | 0 | 0 | 0 | 0 | 0 | 0 | 0 | 0 | 0 | 0 | 0 | 0 | 0 | 0 | 0 |
| *Staurastrum sp.7* | 39.14 | 0 | 0 | 0 | 0 | 0 | 0 | 0 | 0 | 0 | 0 | 0 | 0 | 0 | 0 | 0 | 0 | 39.14 | 0 |
| *Surirella sp.1* | 5.59 | 0 | 0 | 0 | 0 | 0 | 0 | 0 | 0 | 0 | 0 | 5.59 | 0 | 0 | 0 | 0 | 0 | 0 | 0 |
| *Tetraedrum trigonum* | 11.18 | 11.18 | 0 | 0 | 0 | 0 | 0 | 0 | 0 | 0 | 0 | 0 | 0 | 0 | 0 | 0 | 0 | 0 | 0 |
| *Trachelomonas sp.1* | 5.59 | 0 | 0 | 0 | 0 | 5.59 | 0 | 0 | 0 | 0 | 0 | 0 | 0 | 0 | 0 | 0 | 0 | 0 | 0 |
| *Trachelomonas sp.2* | 11.18 | 0 | 0 | 0 | 0 | 0 | 0 | 5.59 | 0 | 0 | 0 | 0 | 0 | 0 | 5.59 | 0 | 0 | 0 | 0 |
| *Urosolenia sp.1* | 11.18 | 11.18 | 0 | 0 | 0 | 0 | 0 | 0 | 0 | 0 | 0 | 0 | 0 | 0 | 0 | 0 | 0 | 0 | 0 |
| *Westella botryoides* | 44.73 | 0 | 0 | 0 | 0 | 0 | 0 | 0 | 39.14 | 0 | 5.59 | 0 | 0 | 0 | 0 | 0 | 0 | 0 | 0 |

**R PROGRAM SCRIPTS**

**1. Taxonomic Resolution**

**For phytoplankton, periphyton, zooplankton and fish data**

install.packages("vegan")

library(vegan)

# importing files and standardization

sp<-read.table("sp.txt",header=TRUE)

sp<-t(sp) # Sampling points must be on the lines

sp<-decostand(sp, method = "hellinger")

genus<-read.table("genus.txt",header=TRUE)

genus<-t(genus) # Sampling points must be on the lines

genus<-decostand(genus, method = "hellinger")

family<-read.table("family.txt",header=TRUE)

family<-t(family) # Sampling points must be on the lines

family<-decostand(family, method = "hellinger")

order<-read.table("order.txt",header=TRUE)

order<-t(order) #Sampling points must be on the lines

order<-decostand(order, method = "hellinger")

class<-read.table("class.txt",header=TRUE)

class<-t(class) # Sampling points must be on the lines

class<-decostand(class, method = "hellinger")

# MANTEL

# Building a Bray Curtis distance matrix

braysp<-vegdist(sp, "bray")

braygenus<-vegdist(genus, "bray")

brayfamily<-vegdist(family, "bray")

brayorder<-vegdist(order, "bray")

brayclass<-vegdist(class, "bray")

# correlate the matrices

species_genus<-mantel(braysp, braygenus, method="pearson", permutations=10000)

species_family<-mantel(braysp, brayfamily, method="pearson", permutations=10000)

species_order<-mantel(braysp, brayorder, method="pearson", permutations=10000)

species_class<-mantel(braysp, brayclass, method="pearson", permutations=10000)

# MNDS

sp.nmds <- metaMDS(sp, distance="bray", k=2)

summary(sp.nmds)

axis.sp<-sp.nmds$points

genus.nmds <- metaMDS(genus, distance="bray", k=2)

summary(genus.nmds)

axis.genus<-genus.nmds$points

family.nmds <- metaMDS(family, distance="bray", k=2)

summary(family.nmds)

axis.family<-family.nmds$points

order.nmds <- metaMDS(order, distance="bray", k=2)

summary(order.nmds)

axis.order<-order.nmds$points

class.nmds <- metaMDS(class, distance="bray", k=2)

summary(class.nmds)

axis. class<-class.nmds$points

#PROCRUSTES

sp_genus<-protest(axis.sp, axis.genus, scores = "sites", permutations = 1000)

sp_family<-protest(axis.sp,axis.family, scores = "sites", permutations = 1000)

sp_order<-protest(axis.sp, axis.order, scores = "sites", permutations = 1000)

sp_class<-protest(axis.sp, axis. class, scores = "sites", permutations = 1000)

########################

**2. Numerical Resolution**

**For phytoplankton, periphyton, zooplankton and fish data**

library(vegan)

# importing files and standardization

sp.abundance<-read.table("sp.abundance.txt", header = TRUE) #data of the species abundance

sp.abundance<-decostand(sp.abundance, method = "hellinger")

sp.ocurrence<-read.table("sp.ocurrence.txt", header = TRUE) # data of the species occurrence

#MANTEL

# Building a Bray Curtis distance matrix

sp.abundance.matrix<-vegdist(sp.abundance, "bray")

sp.occurrence.matrix<-vegdist(sp.occurrence, "jaccard")

# correlate the matrices

abundance_ocurrence<-mantel(sp.abundance.matrix, sp.ocurrence.matrix, method="pearson", permutations=10000)

#NMDS

sp.abundance.nmds <- metaMDS(sp.abundance, distance="bray", k=2)

summary(sp.abundance.nmds)

axis.sp.abundance<-sp.abundance.nmds$points

sp.occurrence.nmds <- metaMDS(sp.occurrence, distance="jaccard", k=2)

summary(sp.occurrence.nmds)

axis.sp.occurrence<-sp.occurrence.nmds$points

#PROCRUSTES

abundance_occurrence<-protest(axis.sp.abundance, axis.sp.occurrence, scores = "sites", permutations = 1000)

#############################

**3. Cross Taxa**

**Biological Groups**

library(vegan)

# importing files and standardization

fish<-read.table("fish.txt",header = TRUE) # data of the abundance of fish species

fish<-t(fish) # Sampling points must be on the lines

fish<-decostand(fish, method = "hellinger")

phytoplankton<-read.table("phytoplankton.txt",header = TRUE) # data of the abundance of phytoplankton species

phytoplankton<-t(phytoplankton) # Sampling points must be on the lines

phytoplankton<-decostand(phytoplankton, method = "hellinger")

periphyton<-read.table("periphyton.txt",header = TRUE) # data of the abundance of periphyton species

periphyton<-t(periphyton) # Sampling points must be on the lines

periphyton<-decostand(periphyton, method = "hellinger")

zooplankton<-read.table("zooplankton.txt",header = TRUE) # data of the abundance of zooplankton species

zooplankton<-t(zooplankton) # Sampling points must be on the lines

zooplankton<-decostand(zooplankton, method = "hellinger")

# MANTEL

# Building a Bray Curtis distance matrix

brayfish<-vegdist(fish, "bray")

brayphytoplankton<-vegdist(phytoplankton, "bray")

brayperiphyton<-vegdist(periphyton, "bray")

brayzooplankton<-vegdist(zooplankton, "bray")

# correlate the matrices

fish_phytoplankton<-mantel(brayfish, brayphytoplankton, method="pearson", permutations=10000)

fish_periphyton<-mantel(brayfish, brayperiphyton, method="pearson", permutations=10000)

fish_zooplankton<-mantel(brayfish, brayzooplankton, method="pearson", permutations=10000)

phytoplankton_periphyton<-mantel(brayphytoplankton, brayperiphyton, method="pearson", permutations=10000)

phytoplankton_zooplankton<-mantel(brayphytoplankton, brayzooplankton, method="pearson", permutations=10000)

zooplankton_periphyton<-mantel(brazooplankton, brayperiphyton, method="pearson", permutations=10000)

# MNDS

fish.nmds <- metaMDS(fish, distance="bray", k=2)

summary(fish.nmds)

axis.fish<-fish.nmds$points

phytoplankton.nmds <- metaMDS(phytoplankton, distance="bray", k=2)

summary(phytoplankton.nmds)

axis.phytoplankton<-phytoplankton.nmds$points

periphyton.nmds <- metaMDS(periphyton, distance="bray", k=2)

summary(periphyton.nmds)

axis.periphyton<-periphyton.nmds$points

zooplankton.nmds <- metaMDS(zooplankton, distance="bray", k=2)

summary(zooplankton.nmds)

axis.zooplankton<-zooplankton.nmds$points

#PROCRUSTES

fish_phytoplankton<-protest(axis.fish, axis.phytoplankton, scores = "sites", permutations = 1000)

fish_periphyton<-protest(axis.fish,axis.periphyton, scores = "sites", permutations = 1000)

fish_zooplankton<-protest(axis.fish, axis.zooplankton, scores = "sites", permutations = 1000)

phytoplankton_periphyton<-protest(axis.phytoplankton, axis. periphyton, scores = "sites", permutations = 1000)

phytoplankton_zooplankton<-protest(axis.phytoplankton, axis. zooplankton, scores = "sites", permutations = 1000)

zooplankton_periphyton<-protest(axis.zooplankton, axis. periphyton, scores = "sites", permutations = 1000)

**Trophic Groups**

library(vegan)

# importing files and standardization

fish<-read.table("fish.txt",header = TRUE) # data of the abundance of fish species

fish<-t(fish) # Sampling points must be on the lines

fish<-decostand(fish, method = "hellinger")

phytoplankton<-read.table("phytoplankton.txt",header = TRUE) # data of the abundance of phytoplankton species

phytoplankton<-t(phytoplankton) # Sampling points must be on the lines

phytoplankton<-decostand(phytoplankton, method = "hellinger")

periphyton<-read.table("periphyton.txt",header = TRUE) # data of the abundance of periphyton species

periphyton<-t(periphyton) # Sampling points must be on the lines

periphyton<-decostand(periphyton, method = "hellinger")

zooplankton<-read.table("zooplankton.txt",header = TRUE) # data of the abundance of zooplankton species

zooplankton<-t(zooplankton) # Sampling points must be on the lines

zooplankton<-decostand(zooplankton, method = "hellinger")

carnivorous.fish<-read.table("carnivorous_fish.txt",header = TRUE)

carnivorous.fish <-t(carnivorous.fish) # Sampling points must be on the lines

carnivorous.fish <-decostand(carnivorous.fish, method = "hellinger")

herbivorous.fish<-read.table("herbivorous_fish.txt",header = TRUE)

herbivorous.fish <-t(herbivorous.fish) # Sampling points must be on the lines

herbivorous.fish <-decostand(herbivorous.fish, method = "hellinger")

herbivorous.zooplankton <-read.table("herbivorous_zooplankton.txt",header = TRUE)

herbivorous.zooplankton<-t(herbivorous.zooplankton) # Sampling points must be on the lines

herbivorous.zooplankton <-decostand(herbivorous.zooplankton, method = "hellinger")

# MANTEL

# Building a Bray Curtis distance matrix

brayphytoplankton<-vegdist(phytoplankton, "bray")

brayperiphyton<-vegdist(periphyton, "bray")

brayzooplankton<-vegdist(zooplankton, "bray")

brayfishC<-vegdist(carnivorous.fish, "bray")

brayfishH<-vegdist(herbivorous.fish, "bray")

brayzooplanktonH<-vegdist(herbivorous.zooplankton, "bray")

# correlate the matrices

herbivorous.fish_phytoplankton<-mantel(brayfishH, brayphytoplankton, method="pearson", permutations=10000)

herbivorous.fish_periphyton<-mantel(brayfishH, brayperiphyton, method="pearson", permutations=10000)

carnivorous.fish_zooplankton<-mantel(brayfishC, brayzooplankton, method="pearson", permutations=10000)

phytoplankton_zooplankton.herbivorous<-mantel(brayphytoplankton, brayzooplanktonH, method="pearson", permutations=10000)

periphyton_zooplankton.herbivorous<-mantel(brayperiphyton, brayzooplanktonH, method="pearson", permutations=10000)

phytoplankton_periphyton<-mantel(brayphytoplankton, brayperiphyton, method="pearson", permutations=10000)

**4. Relationship with the environment and spatial distances**

**#Repeat the analysis separately for each taxonomic level of phytoplankton, periphyton, zooplankton and fish data**

library(vegan)

library(usdm)

library(spdep)

library(ncf)

library(adespatial)

#SPATIAL FILTERS CONSTRUCTION

coordinates<-read.table("coordinates.txt",header=TRUE) # file with the latitude and longitude of the sampling points

distance.matrix<-vegdist(coordinates,method="euclid")

filters<-pcnm(distance.matrix)

filters.pcnm<-filters$vectors

# SPATIAL FILTERS SELECTION

global.model<-rda(data, filters.pcnm)

p.global.model<-anova(global.model, step=10000)

R².global.model<-RsquareAdj(global.model)

filters.sel<-forward.sel(data, filters.pcnm, nperm = 1000, adjR2thresh = R².global.model ,alpha = 0.05)# spatial filters selection. Data represents abundance each taxonomic level (species, genus, family, order and class) of phytoplankton, periphyton, zooplankton and fish. During the selection process, the selection stops if (1) the next selected variables is not significant (> 0.05), and (2) if the R2adj of the model including this variable exceeds the R2adj of the global model.

# ENVIRONMENTAL VARIABLES SELECTION

environment<-read.table("environment.txt",header=TRUE)

environment.VIF.sel<-vifcor(environment, th=0.5)

global.model<-rda(data, environment.VIF.sel)

p.global.model<-anova(global.model, step=10000)

R².global.model <-RsquareAdj(global.model)

environment.sel<-forward.sel(data, environment, nperm = 1000, adjR2thresh = R².global.model, alpha = 0.05) )# environmental variables selection. Data represents abundance each taxonomic level (species, genus, family, order and class) of phytoplankton, periphyton, zooplankton and fish. During the selection process, the selection stops if (1) the next selected variables is not significant (> 0.05), and (2) if the R2adj of the model including this variable exceeds the R2adj of the global model.

RDA

data<-read.table("data.txt",header=FALSE)# data represents abundance each taxonomic level (species, genus, family, order and class) of phytoplankton, periphyton, zooplankton and fish.

data<-decostand(data, "hellinger")

RDA<-varpart(data, environment.sel, filters.sel, data=a)

plot(RDA)

# Anova

siga<-rda(data, environment.sel, filters.sel)

sigb<-rda(data, filters.sel, environment.sel)

plot(siga)

plot(sigb)

A<-anova(siga, step=10000)

B<-anova(sigb, step=10000)

OR

analysis<-rda(data, environment.sel)

p<-anova(analysis, step=10000)

R²adj<-RsquareAdj(analysis)

OR

analysis<-rda(data, filters.sel)

p<-anova(analysis, step=10000)

R²adj<-RsquareAdj(analysis)
